# Lysosomal gene *Hexb* displays haploinsufficiency in a knock-in mouse model of Alzheimer’s disease

**DOI:** 10.1016/j.ibneur.2022.01.004

**Published:** 2022-01-20

**Authors:** Lauren S. Whyte, Célia Fourrier, Sofia Hassiotis, Adeline A. Lau, Paul J. Trim, Leanne K. Hein, Kathryn J. Hattersley, Julien Bensalem, John J. Hopwood, Kim M. Hemsley, Timothy J. Sargeant

**Affiliations:** a The University of Adelaide, School of Medicine, North Terrace, Adelaide, SA, Australia; b Lysosomal Health in Ageing, Hopwood Centre for Neurobiology, Lifelong Health Theme, South Australian Health and Medical Research Institute, North Terrace, Adelaide, SA, Australia; c Childhood Dementia Research Group, Hopwood Centre for Neurobiology, Lifelong Health Theme, South Australian Health and Medical Research Institute, North Terrace, Adelaide, SA, Australia; d Proteomics, Metabolomics and MS-Imaging Core Facility, South Australian Health and Medical Research Institute, North Terrace, Adelaide, SA, Australia; e Hopwood Centre for Neurobiology, Lifelong Health Theme, South Australian Health and Medical Research Institute, North Terrace, Adelaide, SA, Australia

**Keywords:** Aβ, amyloid beta, AD, Alzheimer’s disease, APP, amyloid precursor protein, CD68, cluster of differentiation 68, ELISA, enzyme-linked immunosorbent assay, GFAP, glial fibrillary acidic protein, HEXB, β-hexosaminidase β subunit, IBA1, ionised calcium binding adaptor molecule 1, IL, interleukin, LAMP1, lysosome associated membrane protein 1, LSDs, lysosomal storage disorders, PBS, phosphate buffered saline, TREM2, triggering receptor expressed on myeloid cells 2, Alzheimer’s disease, Dementia, Knock-in, *App*^*NL-G-F/NL-G-F*^, Lysosome, β-hexosaminidase, *Hexb*

## Abstract

Lysosomal network abnormalities are an increasingly recognised feature of Alzheimer’s disease (AD), which appear early and are progressive in nature. Sandhoff disease and Tay-Sachs disease (neurological lysosomal storage diseases caused by mutations in genes that code for critical subunits of β-hexosaminidase) result in accumulation of amyloid-β (Aβ) and related proteolytic fragments in the brain. However, experiments that determine whether mutations in genes that code for β-hexosaminidase are risk factors for AD are currently lacking. To determine the relationship between β-hexosaminidase and AD, we investigated whether a heterozygous deletion of *Hexb*, the gene that encodes the beta subunit of β-hexosaminidase, modifies the behavioural phenotype and appearance of disease lesions in *App*^*NL-G-F/NL-G-F*^*(App*^*KI/KI*^*)* mice. *App*^*KI/KI*^ and *Hexb*^*+/-*^ mice were crossed and evaluated in a behavioural test battery. Neuropathological hallmarks of AD and ganglioside levels in the brain were also examined. Heterozygosity of *Hexb* in *App*^*KI/KI*^ mice reduced learning flexibility during the Reversal Phase of the Morris water maze. Contrary to expectation, heterozygosity of *Hexb* caused a small but significant decrease in amyloid beta deposition and an increase in the microglial marker IBA1 that was region- and age-specific. *Hexb* heterozygosity caused detectable changes in the brain and in the behaviour of an AD model mouse, consistent with previous reports that described a biochemical relationship between HEXB and AD. This study reveals that the lysosomal enzyme gene *Hexb* is not haplosufficient in the mouse AD brain.

## Highlights

• The *App* NL-G-F Alzheimer mouse has lysosomal defects and stores ganglioside lipids.
• Heterozygous lysosomal *Hexb* did not drive amyloidosis in the *App* NL-G-F mouse.
• Heterozygous *Hexb* on an Alzheimer’s background reduced learning flexibility.
• Heterozygous *Hexb* on a wild-type mouse background produced hypoactivity.

## Introduction

Alzheimer’s disease (AD) is a neurodegenerative disease that is the most common cause of dementia (Goodman et al., 2017). It is characterised by insoluble extracellular amyloid beta (Aβ) plaques and intraneuronal tangles of phosphorylated tau (Bloom, 2014), features it shares with lysosomal storage disorders (LSDs) (Whyte et al., 2017). LSDs are caused by the deficiency of an enzyme or protein required for lysosomal function (Filocamo and Morrone, 2011). Many LSDs have a severe neurodegenerative phenotype (Platt et al., 2012, Wraith, 2004): for example, Sandhoff disease is caused by a mutation in the *HEXB* gene (O'Dowd et al., 1986, O'Dowd et al., 1985) which encodes the beta subunit of β-hexosaminidase and leads to developmental regression after the first few months of life, seizures, and death in early childhood (Bley et al., 2011, Smith et al., 2012b). Sandhoff disease is characterised by primary storage of GM2, a ganglioside that is also elevated in AD cerebral cortex (Kracun et al., 1992, Kracun et al., 1991, Molander-Melin et al., 2005). The *Hexb*^*-/-*^ Sandhoff mouse model (Sango et al., 1995) and other mouse models of neurodegenerative LSDs share other features with AD, such as accumulation of amyloid precursor protein (APP) C-terminal fragments, Aβ, and phosphorylated tau in the brain (Annunziata et al., 2013, Beard et al., 2017, Boland et al., 2010, Jin et al., 2004, Keilani et al., 2012, Ohmi et al., 2011, Xu et al., 2014). Intriguingly, *HEXB* has recently been identified as a gene associated with AD (Sierksma et al., 2020), and increasing β-hexosaminidase activity improves the behavioural phenotype in the Dutch APP^E693Q^ mouse (Knight et al., 2015).

Most LSDs, including Sandhoff disease, are inherited in an autosomal recessive manner. Heterozygotes can occur at frequencies of up to 1:15 in certain populations (Fitterer et al., 2014) and have traditionally been considered asymptomatic carriers. However, there is increasing evidence of pathophysiology in heterozygotes who carry a loss-of-function lysosomal gene allele: for example, increased age-specific risk of Parkinson’s disease for Gaucher carriers (Alcalay et al., 2014), development of frontotemporal dementia in carriers of progranulin-associated neuronal ceroid lipofuscinosis (Smith et al., 2012a), and significant disease burden and impaired quality of life in female carriers of X-linked Fabry disease (Wang et al., 2007). These relationships demonstrate that many lysosomal genes could be haploinsufficient in the context of age-related neurological disease and could be risk factors for AD.

Further supporting the importance of the lysosomal network (comprising endo-lysosomal and autophagic pathways) to age-related neurological disease, its dysfunction is also a feature of AD (Cataldo et al., 2000, Nixon, 2017, Nixon and Cataldo, 2006, Peric and Annaert, 2015, Pickford et al., 2008, Whyte et al., 2017). Examination of the AD brain reveals enlarged rab5-positive endosomes (Cataldo et al., 2000, Nixon, 2017) and dystrophic axons filled with lysosomal network cargo vesicles around Aβ plaques (Nixon et al., 2005, Terry et al., 1964). Lysosomal proteins are observed histologically at most Aβ plaques (Barrachina et al., 2006, Bernstein et al., 1989; Cataldo and Nixon, 1990; Cataldo et al., 1991; Hassiotis et al., 2018). Furthermore, dysfunctional lysosomal network compartments demonstrate intracellular Aβ accumulation many years before extracellular Aβ is deposited (Takahashi et al., 2002, Yu et al., 2005). Genetic variation in lysosomal network genes has also been associated with AD in genome-wide association studies (Gao et al., 2018, Lambert et al., 2013).

Based on the shared neuropathological links between AD and LSDs, particularly Sandhoff disease, we hypothesised that heterozygous mutation of *Hexb* exacerbates disease signs in AD. To address this, we crossed *Hexb*^*+/-*^ mice (Sango et al., 1995) with the knock-in *App*^*NL-G-F/NL-G-F*^ AD mouse model (Saito et al., 2014), which we have previously shown to exhibit lysosomal network dysfunction, including abnormal β-hexosaminidase activity in brain homogenates (Whyte et al., 2020).

## Experimental procedures

Additional details are in the Supplementary Information.

### Animals

*App*^*NL-G-F/NL-G-F*^ (hereafter referred to as *App*^KI/KI^) male founder mice were obtained from RIKEN (Saito et al., 2014) on a C57BL/6 J background and a breeding colony established at SAHMRI following re-derivation. *Hexb*^*+/-*^ mice (B6;129S4-*Hexb*^*tm1Rlp*^/J (Sango et al., 1995)) were purchased from The Jackson Laboratory (stock number #002914; Bar Harbor, ME, USA). *App*^KI/KI^ mice were crossed with *Hexb*^*+/-*^ mice and F3 progeny aged up to 46 weeks (n = 240; Supplementary Fig. 1). All animal experimentation was approved by the SAHMRI (SAM129) and University of Adelaide (M-2015-082) Animal Ethics Committees and conducted according to the National Health and Medical Research Council’s *Australian Code for the Care and Use of Animals for Scientific Purposes (8*^*th*^ *edition)*.

### Genotyping

Genomic DNA was prepared from ear notches from mice by incubating with 10% (w/v) chelex at 100^o^C for 20 min. Samples were centrifuged at 15,800 g for 2 min prior to sampling the supernatant for genotyping. Genotyping was carried out using the following primers: 5’-ATCTCGGAAGTGAAGATG-3′, 5’-ATCTCGGAAGTGAATCTA-3′, 5’-TGTAGATGAGAACTTAAC-3′ and 5’-CGTATAATGTATGCTATACGAAG-3′ (for the knocked-in *App* locus) and 5’-ATCTGGACGAAGAGCATCAG-3’, 5’-TAGACTGCTTTGGAAACTGC-3’ and 5’-TCAGGAAGGAAGTGTCTCAC-3’ (for the *Hexb* KO locus). Reactions were performed with HotStar Taq Master Mix. Two microlitres of chelex supernatant that contained genomic DNA was added for each reaction. PCR cycling conditions consisted of an initial activation step at 95°C for 15 min, followed by 35 cycles of: denaturation at 95°C for 30 s, primer annealing at 55°C or 60°C for *App* and *Hexb* respectively for 30 s, and extension at 72°C for 1 min, with a final extension at 72°C for 10 min. PCR products were run on a 3% (w/v) or a 2% (w/v) agarose gel for *App* and *Hexb* genotyping respectively, and the genotype of each animal determined after DNA staining.

### Behaviour

Two cohorts of male mice were tested in a behavioural test battery commencing at either 26 or 39 to 40 weeks of age (n = 15 male mice/genotype) by an experimenter blinded to genotype.

#### Y-maze

At 26 (n = 60 mice total) or 39 to 40 weeks (n = 60 mice total) of age mice were tested for working memory in the Y-maze spontaneous alternation test as described previously (Saito et al., 2014, Whyte et al., 2018). The number of arm entries, total path length, and average speed was recorded, and percentage alternation was calculated. One mouse from the 39- to 40-week cohort was excluded from analysis because it only entered one arm during the trial, making it impossible to assess percentage alternation.

#### Open Field and Novel Object Recognition tests

Activity levels were assessed in an open field at 27 or 40 to 41 weeks as described previously (Whyte et al., 2018). The Open Field test served as the habituation phase for the Novel Object Recognition test, which was performed using a method adapted from (Leger et al., 2013) and described in (Whyte et al., 2018). Five mice from the 27-week cohort and seven mice from the 40- to 41-week cohort were excluded from analysis prior to un-blinding due to < 20 s interaction time with objects on Days 2 and/or 3. Such a criterion ensures a similar exploration time of the two objects and between animals independently of their individual exploratory activity (Leger et al., 2013).

#### Morris water maze

Mice were subsequently tested for learning and spatial memory using the Morris water maze from 28 to 30 weeks or 42 to 44 weeks of age, as previously described (Hemsley et al., 2009, Whyte et al., 2018), with the addition of a Reversal Phase to test for cognitive flexibility involving a complete replication of the Acquisition Phase, except that the platform was positioned 35 cm from the wall in the opposite (SE) quadrant. On the day following the Reversal Phase, a Reversal Probe test was conducted. The platform was removed from the pool and each mouse was given a single 90-s swim commencing from the NW quadrant. Visual testing was also performed following completion of the Reversal Phase by determining the time taken to find a visible platform.

### Tissue collection

All mice were humanely culled at 4, 8, 16, 32 or 46 weeks via carbon dioxide asphyxiation (Supplementary Fig. 1). Mice for biochemical analysis (n = 5–10/age/genotype) were transcardially perfused with ice-cold phosphate buffered saline (PBS) to remove blood. Brains were removed, divided along the midline, and slices taken 2–4 mm and 4–6 mm caudal of the olfactory bulb. Cortical tissue was dissected from both slices for ELISA and enzyme activity/ganglioside analyses, respectively. Hippocampal tissue was dissected from the latter slice of both hemispheres for ELISA. Tissue was snap-frozen in liquid nitrogen and stored at − 80 °C. Mice for immunohistochemistry (n = 5/age/genotype) were transcardially perfused with ice-cold PBS followed by 4% (w/v) paraformaldehyde in PBS. Brains were post-fixed in 4% (w/v) paraformaldehyde in PBS for seven days and stored in PBS at 4 °C, then embedded in paraffin wax.

### Lysosomal enzyme activity measurement

Cortical tissue was homogenised for assay of cathepsins D/E, β-galactosidase, cathepsins B/L, and β-hexosaminidase activities, as described in Whyte et al., 2020.

### Immunohistochemistry

Six micrometre sagittal sections were cut on a microtome (RM2235 Leica, Wetzlar, Germany) 0.36–0.96 mm lateral from the midline (based on stereotaxic coordinates in a mouse brain atlas (Paxinos and Franklin, 2001)). Paraffin sections were dewaxed and rehydrated prior to antigen retrieval with 90% formic acid (for Aβ) or 10 mM citrate buffer, pH 6.0 (for glial fibrillary acidic protein (GFAP), ionised calcium binding adaptor molecule 1 (IBA1), lysosome-associated membrane protein 1 (LAMP-1), cluster of differentiation 68 (CD68), and trigger receptor expressed on myeloid cells 2 (TREM2)). Synaptophysin required no pre-treatment. Following blocking of non-specific proteins in 10% normal donkey serum, sections were incubated overnight in a humidified chamber at room temperature with primary antibodies: anti-Aβ 1:200, anti-GFAP 1:13,000, anti-IBA1 1:2,000, anti-LAMP-1 1:750, anti-CD68 1:1,000, anti-TREM2 1:500 or anti-synaptophysin 1:20,000. After washing in PBS, sections were labelled with species-specific biotinylated-conjugated secondary antibodies (1:2,000 in PBS) for 1–1.5 h. Sections were washed with PBS, conjugated with avidin (Vectastain Elite ABC Kit [Aβ, GFAP, IBA1] or peroxidase-conjugated streptavidin [LAMP-1, CD68, TREM2, synaptophysin]), then developed using the DAKO DAB+ substrate chromogen system. Following a light counterstain with haematoxylin, sections were dehydrated, cleared, and coverslipped. Please see supplementary information for catalogue numbers.

### Image analysis and quantification of immunohistochemistry

Images were acquired at 40x magnification on a Pannoramic 250 Flash II Slide Scanner and viewed using the Case Viewer Program (v2.1), both from 3D HISTECH (Budapest, Hungary). Thresholding was applied to images in a consistent manner using Fiji software (Schindelin et al., 2012) to calculate the percentage area stained for Aβ, GFAP, IBA1, and LAMP-1. All staining and image analyses were undertaken by an experimenter blinded to genotype and age.

Quantification of synaptophysin, CD68, and TREM2 in *App*^*KI/KI*^*; Hexb*^*+/+*^ (n = 5) and *App*^*KI/KI*^*; Hexb*^*-/+*^ (n = 5) mice aged 16, 32 and 46 weeks (synaptophysin) or 16 and 32 weeks (CD68; TREM2) was undertaken by visualising scanned images of immunohistochemically stained sections in the Case Viewer Program and manually counting specifically defined staining criteria for each marker in each region of interest. The defined staining criteria used in the quantification for each marker was as follows: synaptophysin-positive dystrophies defined as either disorganised processes with/without small spheroid-like swellings at ends of processes or enlarged swollen process discrete areas that were morphologically consistent with the presence of an amyloid plaque; CD68-positive clusters of microglia at presumed plaques; TREM2-positive ‘ring-like’ structures at presumed plaques. The number of synaptophysin-positive dystrophies, CD68-positive clusters, and TREM2-positive structures in each region was determined and data were reported as the average number of synaptophysin-positive dystrophies per area mm^2^, average number of CD68-positive clusters per area mm^2^, and average number of TREM2-positive structures per area mm^2^.

### Aβ ELISA

Tissues (left and right hemisphere combined) were homogenised in lysing matrix D tubes using a Precellys 24 homogenizer (Bertin Technologies, France) for two cycles of 20 s at 6,500 rpm at 4 °C in 50 mM Tris-HCl, pH 7.4, containing 150 mM NaCl with cOmplete EDTA-free protease inhibitor cocktail (Sigma Aldrich) and 1 mM EDTA (tris-buffered saline (TBS); cortical samples) or 0.5 M guanidine/50 mM TBS with cOmplete EDTA-free protease inhibitor cocktail (Sigma Aldrich) and 1 mM EDTA (hippocampal samples). Samples were allowed to rest for 30 s between cycles. For cortical samples, TBS-soluble material was separated from insoluble material in guanidine-HCl (GuHCl fraction) as described previously (Iwata et al., 2004).

Aβ_x-40_ and Aβ_x-42_ (hereafter referred to as Aβ_40_ and Aβ_42_) were quantified in cortical TBS-soluble and GuHCl fractions, as well as hippocampal samples using human/rat β-amyloid (40) and β-amyloid (42) ELISA kits (294-64701, and 292-64501, respectively; FUJIFILM Wako Pure Chemical Corporation). Standard curves were generated using synthetic rat Aβ peptides (*App*^*+/+*^*; Hexb*^*+/+*^ and *App*^*+/+*^*; Hexb*^*+/-*^ samples) and synthetic human Aβ peptides containing the Arctic mutation (*App*^KI/KI^*; Hexb*^*+/+*^ and *App*^*KI/KI*^*; Hexb*^*+/-*^ samples).

### Ganglioside quantitation

Cortical tissues (left hemisphere) were homogenised as described for Aβ ELISAs in 0.02 M Tris, 0.5 M NaCl, pH 7.4. Total protein was determined using a Micro-BCA Protein Assay Kit (23225; Thermo Scientific). Cortical homogenates (100 µg total protein) were then spiked with deuterated (d_3_) internal standards (all d18:1/18:0): 500 ng GM1 (2050; Matreya LLC, PA, USA), 250 ng GM2 (2051; Matreya LLC) and 250 ng GM3 (2052; Matreya LLC). Protein precipitation was performed by adding 750 µL methanol and incubating at − 20 °C for 1 h. Samples were centrifuged at 16,200 g for 10 min to remove precipitate and the supernatant used for quantification. Samples were analysed using an Acquity ultra performance liquid chromatography system (Waters Corporation, Milford, MA, USA) fitted with a BEH C18 2.1 × 50 mm analytical column (Waters Corporation) and coupled to an API4000 Q-trap mass spectrometer (Sciex, Framingham, MA, USA). Liquid chromatography gradient separation of a 10 µL sample injection was performed from starting conditions of 10% mobile phase A (90:10 (H_2_O:MeOH) 1 mM ammonium acetate), 90% mobile phase B (MeOH containing 1 mM ammonium acetate), to 95% mobile phase B over 2.7 min and held for 1.2 min. The column was then washed with 99.9% mobile phase B for 2 min prior to 1 min re-equilibration with 90% mobile phase B. A flow rate of 350 µL/min was used. Peak areas were integrated in Analyst 1.6.2 (Sciex) and normalised to the d_3_ internal standards. Mass spectrometric transitions are listed in Supplementary Table 1.

### Statistics

Statistical analyses were performed using GraphPad Prism v7.02. When investigating the main effects of and interactions between three independent variables (i.e*.* age/time, *App* genotype, and *Hexb* genotype) a two-step analysis was used: first, a three-way ANOVA model was used to investigate the overall effect of age/time, *App* genotype, and *Hexb* genotype, and possible interaction between those three variables. The effect of *App* genotype and *Hexb* genotype and the interaction between *Hexb* and *App* was then investigated for each individual time point with a two-way ANOVA followed by Tukey’s multiple comparison tests when there was a significant App*Hexb interaction. When investigating the main effects of, and interactions between, two independent variables (i.e*. App* genotype and *Hexb* genotype or age and *Hexb* genotype) a two-way ANOVA was used followed by Tukey’s multiple comparison tests when there was a significant interaction between the two factors. All post hoc test results were automatically adjusted for multiple comparisons. One sample t test was used to determine whether group means were statistically different from chance level (i.e*.* 25% or 50%). A p value less than 0.05 was considered statistically significant.

## Results

### Lysosomal enzyme activities are elevated in *App^KI/KI^* mice

**Fig. 1:**
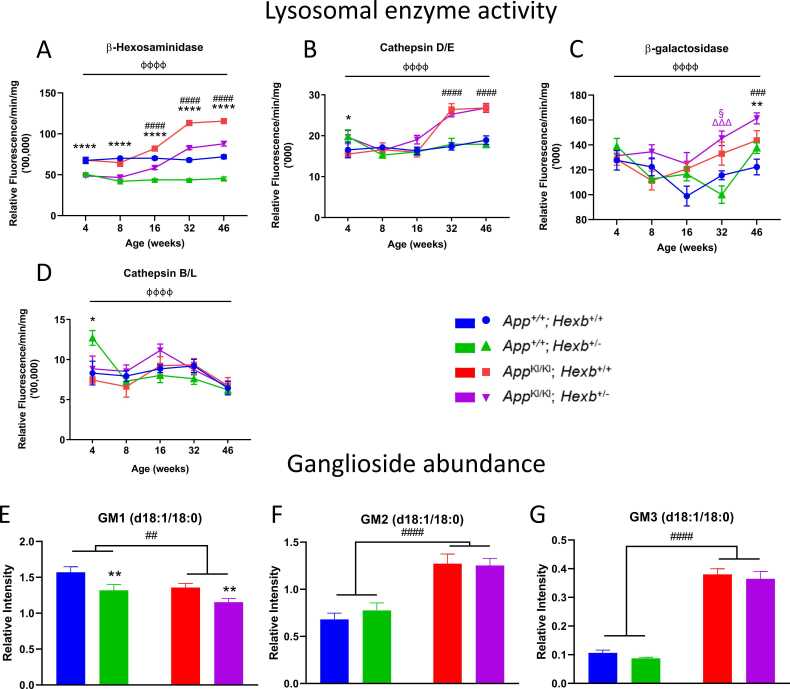
Lysosomal enzyme activities in mouse cortex change in response to *App* and *Hexb* genotype. β-Hexosaminidase (A), cathepsin D/E (B), β-galactosidase (C), and cathepsin B/L (D) activities were measured with fluorogenic substrates in cortical homogenates from *App*^+/+^; *Hexb*^+/+^, *App*^+/+^; *Hexb*^+/-^, *App*^KI/KI^; *Hexb*^+/+^ and *App*^KI/KI^; *Hexb*^+/-^ mice at 4, 8, 16, 32 and 46 weeks. n = 5–10 mice of mixed sex/group; results are mean ± SEM, фффф p < 0.0001 age effect; * p < 0.05, ** p < 0.01, **** p < 0.0001, *Hexb* genotype effect; ### p < 0.001, #### p < 0.0001, *App* genotype effect; § p < 0.05 colour-indicated group vs *App*^+/+^; *Hexb*^+/+^; ΔΔΔ p < 0.001 colour-indicated group vs *App*^+/+^; *Hexb*^+/-^ (2-step analysis: age, *App* genotype and *Hexb* genotype effects and age*App*Hexb interaction analysed with a three-way ANOVA; individual time points were then analysed with a two-way ANOVA followed by Tukey’s multiple comparison tests when App*Hexb interaction p < 0.05). GM1 (E), GM2 (F), and GM3 (G) (d18:1/18:0 species) were measured in cortical homogenates from 46-week-old *App*^+/+^; *Hexb*^+/+^, *App*^+/+^; *Hexb*^+/-^, *App*^KI/KI^; *Hexb*^+/+^, and App^KI/KI^; *Hexb*^+/-^ mice. n = 10 mice of mixed sex/group, error bars = ± SEM^, *^* p < 0.01, *Hexb* genotype effect; ## p < 0.01, #### p < 0.0001, *App* genotype effect (two-way ANOVA).

*App*^*KI/KI*^ mice were crossed with *Hexb*^*+/-*^ mice to generate mice with three familial AD mutations and heterozygous deletion of the *Hexb* gene (*App*^*KI/KI*^*; Hexb*^*+/-*^). To verify *Hexb* heterozygosity we assayed β-hexosaminidase activity in cortical homogenates from *App*^*+/+*^*; Hexb*^*+/+*^, *App*^*+/+*^*; Hexb*^*+/-*^, *App*^*KI/KI*^*; Hexb*^*+/+*^*,* and *App*^*KI/KI*^*; Hexb*^*+/-*^ mice up to 46-weeks of age. As expected, β-hexosaminidase activity was significantly reduced by 25–35% in *Hexb*^*+/-*^ mice when compared with *Hexb*^*+/+*^ mice (Fig. 1A). Conversely, β-hexosaminidase activity was significantly increased by 23–75% from 16 weeks of age in *App*^*KI/KI*^ mice in comparison with *App*^*+/+*^ mice. No significant interaction was found between *Hexb* and *App* genotypes.

Activity of cathepsins D/E was significantly elevated in both *App*^*KI/KI*^*; Hexb*^*+/+*^ and *App*^*KI/KI*^*; Hexb*^*+/-*^ mice compared with *App*^*+/+*^*; Hexb*^*+/+*^ mice (Fig. 1B), however, *Hexb* heterozygosity had no effect. β-galactosidase activity was significantly higher in *App*^*KI/KI*^ mice and *Hexb*^*+/-*^ mice at 46 weeks as a genotype effect measured by two-way ANOVA (Fig. 1C). *Hexb* heterozygosity did not have a major effect on activity of cathepsins B/L (Fig. 1D).

### *Hexb* heterozygosity decreases GM1ganglioside but does not change GM2 and GM3 gangliosides in the *App^KI/KI^* mouse cortex

d18:1/18:0 species of GM1, GM2, and GM3 gangliosides were measured in cortical homogenates from 46-week-old mice. The *App* knock-in allele and the *Hexb* knock-out allele independently reduced GM1 ganglioside (Fig. 1E). However, there were no interactions between the two alleles. GM2 and GM3 d18:1/18:0 were significantly higher in *App*^*KI/KI*^ mice (Fig. 1F, G). However, *Hexb* heterozygosity did not affect the amounts of GM2 (the endogenous substrate of the enzyme that *Hexb* codes for) or GM3 gangliosides.

### *App* knock-in mutations and heterozygosity for *Hexb* induce activity deficits during testing in the Y-maze and Open Field paradigms

All genotypes were phenotypically indistinguishable at birth. *Hexb* heterozygosity did not significantly change body weight, whereas the *App* knock-in mutation decreased body weight gain in females but not in males (Supplementary Fig. 2). To assess the phenotypic effect of *Hexb* heterozygosity in *App*^*KI/KI*^ mice, we performed a behavioural test battery on separate cohorts of mice commencing from 26 or 39 to 40 weeks of age (Supplementary Fig. 3). Activity and working memory, assessed in the Y-maze test, were not affected in *App*^*KI/KI*^*; Hexb*^*+/+*^ mice at 26 weeks of age (Supplementary Fig. 3A-D). However, by 39 to 40 weeks of age, an *App* genotype effect was detected for path length, arm entries, and average speed (Supplementary Fig. 3E-G). Similarly, *Hexb* heterozygosity caused a significant reduction in path length, number of arm entries, and average speed (Supplementary Fig. 2E-G). Contrary to expectation, *App*^*KI/KI*^ mice had increased arm alternation when compared with *App*^*+/+*^ mice (Supplementary Fig. 3H).

**Fig. 2:**
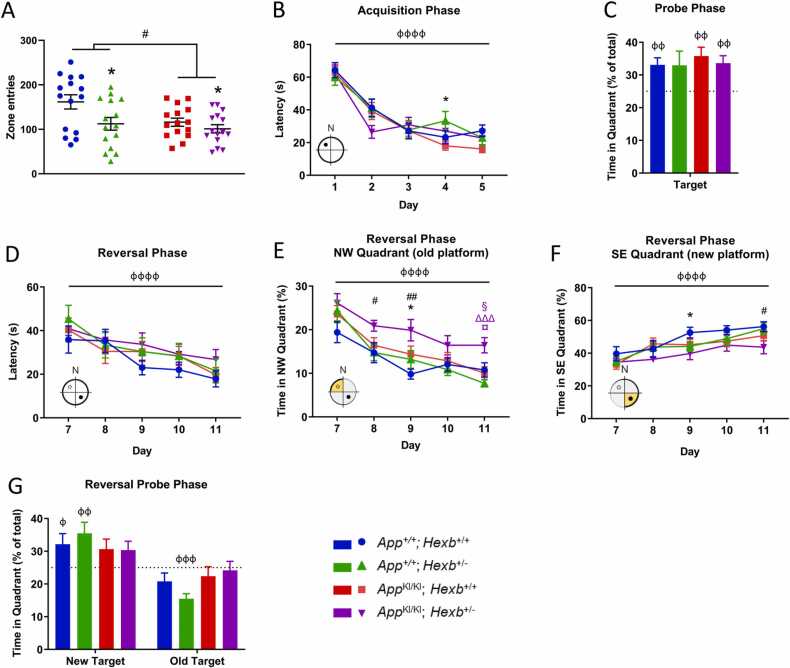
*App* knock-in and *Hexb* heterozygosity cause activity and learning flexibility deficits. Locomotor and exploratory activity were assessed in an Open Field test with male mice at 40 to 41 weeks by measuring zone entries. n = 9–15 male mice/group, error bars = ± SEM, * p < 0.05, *Hexb* genotype effect; # p < 0.05, *App* genotype effect (two-way ANOVA) (A). Male mice were evaluated in the Morris water maze at 42 to 44 weeks of age. Average time to reach the platform in the Acquisition Phase is shown (B). In the Probe Phase the platform was removed and mice were tested in their ability to recall its location. The percentage of time spent in the target quadrant was measured (C; dashed line = chance). The platform was placed in the opposite quadrant of the pool for the duration of the Reversal Phase. Average time to reach the platform (D) and the percentage of swim time spent in the NW (old platform) (E) and SE (new platform) (F) quadrants was recorded. On Day 12 the platform was again removed from the pool and the percentage of time spent in the new target quadrant and the old target quadrant was measured (G; dashed line = chance). n = 15 male mice/group, error bars = ± SEM. B; D-F: фффф p < 0.0001 time effect; * p < 0.05, *Hexb* genotype effect; # p < 0.05, ## p < 0.01, *App* genotype effect; § p < 0.05 colour-indicated group vs *App*^+/+^; *Hexb*^+/+^; ΔΔΔ p < 0.001 colour-indicated group vs *App*^+/+^; *Hexb*^+/-^; ¤ p < 0.05, colour-indicated group vs *App*^KI/KI^; *Hexb*^+/+^; (2-step analysis: time, *App* genotype and *Hexb* genotype effects and time*App*Hexb interaction analysed with a three-way repeated measures ANOVA; individual time points were then analysed with a two-way ANOVA followed by Tukey’s multiple comparison tests when App*Hexb interaction p < 0.05). C; G: ф p < 0.05, фф p < 0.01 compared with chance level (i.e. 25%) (analysed with one sample t-test and two-way ANOVA).

The Open Field test produced similar results to those observed in the Y-maze. In the younger cohort (27 weeks old) there were no significant differences in any of the Open Field parameters measured (Supplementary Fig. 4A-C). By 40 to 41 weeks both *App*^*KI/KI*^ and *Hexb*^*+/-*^ genotypes decreased zone entries compared to *App*^*+/+*^ and *Hexb*^*+/+*^, respectively, (Fig. 2A) with no genotype interaction. This indicates haploinsufficiency for *Hexb*. *App*^*KI/KI*^ mice also had reduced path length and speed (Supplementary Fig. 4E-F). Most of the experimental groups exhibited learning in the Novel Object Recognition test, however, no clear relationship emerged for genotype and recognition memory deficit across the 27- and 40- week time points (Supplementary Fig. 4D,G).

### *App^KI/KI^*; *Hexb^+/-^* mice show a deficit in learning flexibility on the Reversal Phase of the Morris water maze

Spatial memory and learning were assessed in the Morris water maze undertaken at 28 to 30 weeks or 42 to 44 weeks. At both ages all groups exhibited the capacity to use distal cues to learn the location of the hidden platform. There were no significant differences between individual genotypes in latency to reach the platform (Supplementary Fig. 5A,C; Fig. 2B,D) during the Acquisition or Reversal Phases. The only exception was for a *Hexb* effect during the last time point in the Reversal Phase for the 28-week-old animals (Supplementary Fig. 5C) and during Day 4 of learning in the Acquisition Phase for the 42-44-week-old animals (Fig. 2B). In the Probe and Reversal Probe Phases at 28 weeks of age the proportion of time spent in the quadrant housing the platform during the Acquisition and Reversal Phases, respectively, was different from chance for all groups, indicating excellent learning and flexibility (Supplementary Fig. 5B,F). At 42 to 44 weeks, the proportion of time spent by *App*^+/+^; *Hexb*^+/+^, *App*^KI/KI^; *Hexb*^+/+^, and *App*^KI/KI^; *Hexb*^+/-^ mice in the target quadrant during the Probe phase was also statistically different from chance (Fig. 2C). However, in the Reversal Probe phase, *App*^KI/KI^; *Hexb*^+/+^ and *App*^KI/KI^; *Hexb*^+/-^ mice were not statistically different from chance, indicating a deficit in learning flexibility (Fig. 2G).

To examine behavioural flexibility in more detail we measured the proportion of time spent in each quadrant during the Reversal Acquisition Phase. For the 28- to 30-week cohort, there were no differences between genotypes in the proportion of time spent in the quadrant that housed the platform during the previous Acquisition Phase (NW; Supplementary Fig. 5D) or the quadrant housing the new platform (SE; Supplementary Fig. 5E). At 42 to 44 weeks *App*^*KI/KI*^*; Hexb*^*+/-*^ mice spent more time during the Reversal Acquisition Phase in the quadrant that housed the platform during the Acquisition Phase (NW) compared with *App*^*+/+*^*; Hexb*^*+/+*^*, App*^*+/+*^*; Hexb*^*+/-*^, and *App*^*KI/KI*^*; Hexb*^*+/+*^ mice each by post-hoc analysis (Fig. 2E). *Hexb* and *App* genotype effects were detected on Days 9 and 11 during the Reversal Phase, respectively, when time in the new platform quadrant was analysed (Fig. 2F). Swim speed was measured during the Probe Phases, with no significant differences between the genotypes at either age (Supplementary Fig. 6A,B). All mice from both cohorts passed the Visual Phase of the test (data not shown).

### *Hexb* heterozygosity reduces region-specific Aβ in 46-week-old *App^KI/KI^* mice

**Fig. 3:**
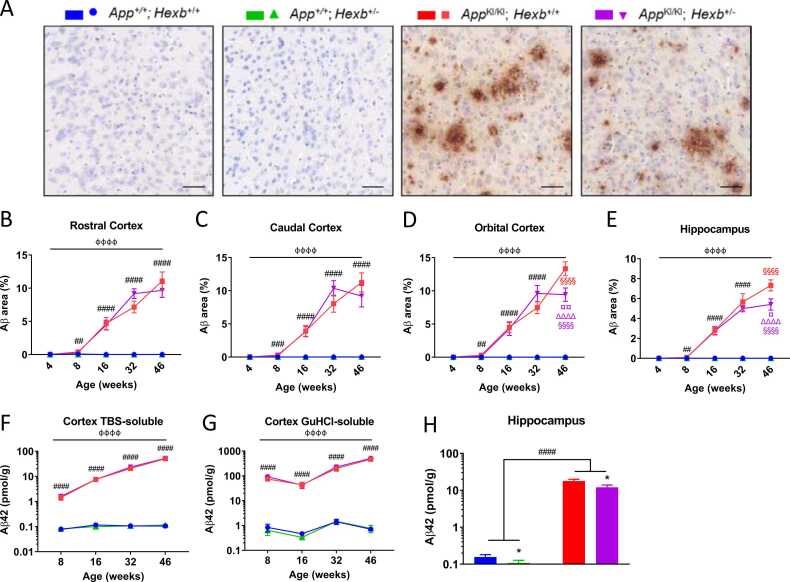
Accumulation of Aβ in the *App* knock-in mouse is decreased in response to *Hexb* heterozygosity. Representative images of Aβ (82E1) staining in 46-week-old mice in the orbital cortex. Scale bars = 50 µm (A). Quantification of the area of Aβ staining in *App*^+/+^; *Hexb*^+/+^, *App*^+/+^; *Hexb*^+/-^, *App*^KI/KI^; *Hexb*^+/+^ and *App*^KI/KI^; *Hexb*^+/-^ mice at 4 , 8 , 16 , 32 and 46 weeks was performed in the rostral cortex, caudal cortex, orbital cortex, and hippocampus, (B-E, n = 5 mice/group, error bars = ± SEM). Aβ42 peptide was quantified by ELISA in TBS-soluble (F) and GuHCl (G) fractions of cortical homogenates from 8-, 16-, 32- and 46-week-old mice and hippocampal homogenates from 46-week-old mice (H), n = 3–9 mice of mixed sex/group, error bars = ± SEM. B-G: фффф p < 0.0001 age effect; ## p < 0.01, ### p < 0.001, #### p < 0.0001, *App* genotype effect; §§§§ p < 0.0001 colour-indicated group vs *App*^+/+^; *Hexb*^+/+^; ¤ p < 0.05, ¤¤ p < 0.01 colour-indicated group vs *App*^KI/KI^; *Hexb*^+/+^; ΔΔΔΔ p < 0.0001 colour indicated group vs *App*^+/+^; *Hexb*^+/-^.(B-G, 2-step analysis: age, *App* genotype and *Hexb* genotype effects and age*App*Hexb interaction analysed with a three-way ANOVA; individual time points were then analysed with a two-way ANOVA followed by Tukey’s multiple comparison tests when App*Hexb interaction p < 0.05; I: two-way ANOVA). H: Two-way ANOVA, #### p < 0.0001, *App* genotype effect; * p < 0.05, *Hexb* genotype effect.

Aβ levels were measured in the brain by immunohistochemistry and ELISA. No Aβ plaques were detected histologically in *App*^*+/+*^*; Hexb*^*+/+*^ or *App*^*+/+*^*; Hexb*^*+/-*^ mice up to 46 weeks of age (Fig. 3A). *App*^*KI/KI*^*; Hexb*^*+/+*^ mice developed plaques by eight weeks of age. Aβ staining increased with age in the *App*^*KI/KI*^ mice, and the percentage area stained became significantly higher than *App*^*+/+*^*; Hexb*^*+/+*^ by eight-weeks in the rostral, intermediate, caudal, and orbital cortices (Fig. 3B-D, Supplementary Fig. 7A), hippocampus (Fig. 3E), and inferior colliculus (Supplementary Fig. 7C). Aβ accumulated more gradually in the thalamus, becoming significantly elevated compared to *App*^*+/+*^ by 16 weeks (Supplementary Fig. 7B). *App*^*KI/KI*^; *Hexb*^*+/-*^ mice exhibited a lower percentage area stained compared with *App*^*KI/KI*^*; Hexb*^*+/+*^ at 46 weeks in the orbital cortex (Fig. 3A, D) and hippocampus (Fig. 3E).

ELISA measurement of Aβ_42_ revealed expected increases in *App*^*KI/KI*^ mice compared with *App*^*+/+*^ mice in cortical TBS-soluble fractions (Fig. 3F) and in cortical GuHCl-soluble fractions (Fig. 3G). Aβ_40_ was also elevated in *App*^*KI/KI*^ mice compared with *App*^*+/+*^ in cortical TBS-soluble fractions (Supplementary Fig. 7D) and in cortical GuHCl-soluble fractions (Supplementary Fig. 7E). There was no overall effect of *Hexb* heterozygosity on Aβ_40_ and Aβ_42_ levels in cortical samples, except in the cortical GuHCl fraction where Aβ_40_ was decreased in *App*^*KI/KI*^; *Hexb*^*+/-*^ compared with *App*^*KI/KI*^; *Hexb*^*+/+*^ (Supplementary Fig. 7E). We also measured Aβ_42_ and Aβ_40_ in hippocampal homogenates from 46-week-old mice. Consistent with the immunohistochemical data, we detected that *App* knock-in increased and that *Hexb* heterozygosity decreased the amount of Aβ_42_ in 46-week hippocampal homogenates (Fig. 3H). We detected an *App* genotype effect but not a *Hexb* genotype effect on the amount of Aβ_40_ in 46-week hippocampal homogenates (Supplementary Fig. 7F).

### *Hexb* heterozygosity increases IBA1staining in the *App^KI/KI^* mouse brain at 32 weeks of age

**Fig. 4:**
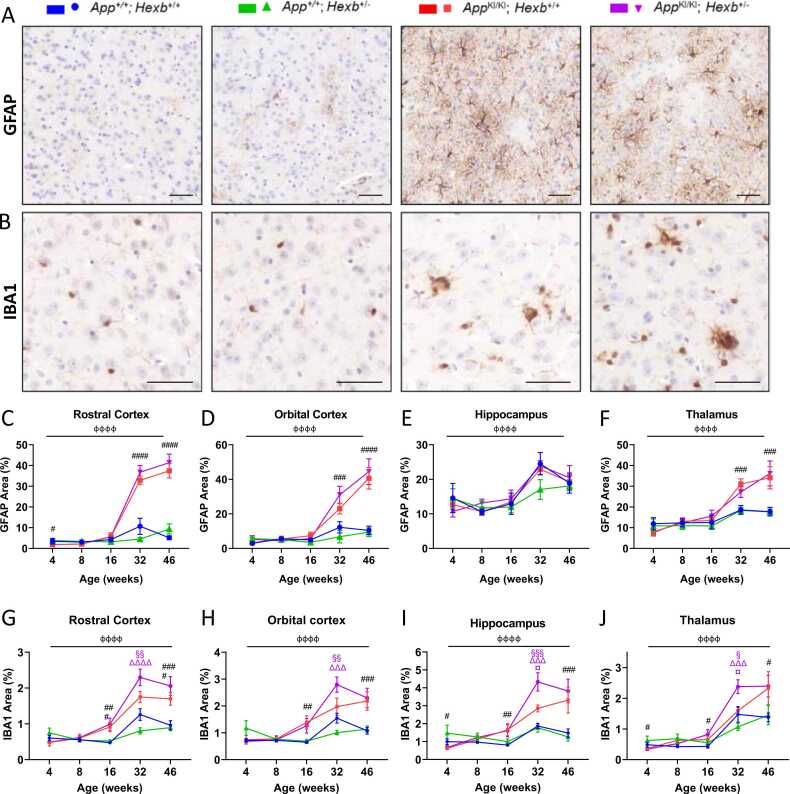
*Hexb* heterozygosity increases staining for microglial marker IBA1 in *App* knock-in mice. Representative images of GFAP (A) and IBA1 (B) staining in 46-week-old mice in the orbital cortex. Scale bars = 50 µm. Quantification of the area of GFAP and IBA1 staining in *App*^+/+^; *Hexb*^+/+^, *App*^+/+^; *Hexb*^+/-^, *App*^KI/KI^; *Hexb*^+/+^ and *App*^KI/KI^; *Hexb*^+/-^ mice at 4 , 8 , 16 , 32 and 46 weeks was performed in the rostral cortex (C, G), orbital cortex (D, H), hippocampus (E, I), and thalamus (F, J), n = 5 mice of mixed sex/group, error bars = ± SEM, фффф p < 0.0001 age effect; # p < 0.05, ## p < 0.01, ### p < 0.001, #### p < 0.0001, *App* genotype effect; § p < 0.05, §§ p < 0.01, §§§ p < 0.001 colour-indicated group vs *App*^+/+^; *Hexb*^+/+^; ¤ p < 0.05, colour-indicated group vs *App*^KI/KI^; *Hexb*^+/+^; ΔΔΔ p < 0.001, ΔΔΔΔ p < 0.0001, colour indicated group vs *App*^+/+^; *Hexb*^+/-^ (2-step analysis: age, *App* genotype and *Hexb* genotype effects and age*App*Hexb interaction analysed with a three-way ANOVA; individual time points were then analysed with a two-way ANOVA followed by Tukey’s multiple comparison tests when App*Hexb interaction p < 0.05).

Low levels of GFAP-positive astrocyte staining were observed in *App*^*+/+*^*; Hexb*^*+/+*^ and *App*^*+/+*^*; Hexb*^*+/-*^ brains at all ages examined (Fig. 4A, C-F; Supplementary Fig. 8A-C). GFAP reactivity in *App*^KI/KI^ mice was significantly higher than *App*^*+/+*^ mice in the rostral cortex (Fig. 4C), the orbital cortex (Fig. 4D), the intermediate cortex (Supplementary Fig. 8A), caudal cortex (Supplementary Fig. 8B), and thalamus (Fig. 4F) from 32 weeks of age. No *Hexb* genotype effects were observed, apart from in the inferior colliculus at 46 weeks, where the *App*^*KI/KI*^; *Hexb*^*+/-*^ displayed higher GFAP levels than the other groups (Supplementary Fig. 8C). No group difference in GFAP reactivity was observed in the hippocampus (Fig. 4E).

*App*^*+/+*^*; Hexb*^*+/+*^ and *App*^*+/+*^*; Hexb*^*+/-*^ brains showed low levels of staining for the microglial/macrophage marker, IBA1 (Fig. 4B, G-J; Supplementary Fig. 8D-F). *App*^*KI/KI*^*; Hexb*^*+/+*^ and *App*^*KI/KI*^; *Hexb*^*+/-*^ mice developed clusters of IBA1-positive microglia from 16 weeks. There were statistically significant *App* genotype effects in the percentage area of positive IBA1 staining in all regions examined (Fig. 4G-J; Supplementary Fig. 8D-F). Although there was significantly more IBA1 staining in the rostral cortex, orbital cortex, hippocampus, thalamus (Fig. 4G-J), and inferior colliculus (Supplementary Fig. 8F) in *App*^*KI/KI*^; *Hexb*^*+/-*^ mice when compared with *App*^*KI/KI*^*; Hexb*^*+/+*^ mice at 32 weeks, the difference did not persist at 46 weeks.

### Markers of tissue damage and inflammation increase over time in *App^KI/KI^* mice

After observing changes in microglial distribution by staining for IBA1 at 32 weeks, we explored additional markers that revealed AD-related tissue damage. As previously documented (Whyte et al., 2020), staining for LAMP1 showed accumulation of lysosomal cargo in a pattern consistent with amyloid plaques (Supplementary Fig. 9A). LAMP1 staining increased in *App* knock-in mice but did not change depending on *Hexb* status in any region analysed (Supplementary Fig. 9B-H). Staining for synaptophysin, a presynaptic marker, revealed loss of synaptic staining in regions where plaques had grown as well as dystrophic axonal compartments that were usually associated with plaques (Supplementary Fig. 10A). While differences in the number of dystrophies over time were not observed in the rostral cortex, increasing numbers of dystrophies were observed over time in the intermediate and caudal cortices. In the caudal cortex, *App*^*KI/KI*^; *Hexb*^*+/-*^ mice had more dystrophies than *App*^*KI/KI*^; *Hexb*^*+/+*^ mice, which was determined by analysis of genotype effect (taking all time points into account) using a two-way ANOVA (Supplementary Fig. 10B-D).

Analysis of the microglial markers CD68 and TREM2 in *App*^*KI/KI*^; *Hexb*^*+/+*^ and *App*^*KI/KI*^; *Hexb*^*+/-*^ mice showed increases between 16 and 32 weeks of age, however, no differences were detected between these two genotypes at these two ages. (Supplementary Figs. 11–12). Interestingly, TREM2 was not detected in *App* knock-in mice at 16 weeks of age (Supplementary Fig. 12). Exploration of proinflammatory cytokines (IL-1β, IL-6, and TNF-α) by ELISA in brain lysates did not reveal any disease-specific changes in abundance at 16 or 32 weeks of age (Supplementary Fig. 13).

## Discussion

*Hexb*^*-/-*^ mice have previously been shown to accumulate APP C-terminal fragments, Aβ, and phosphorylated tau in the brain (Boland et al., 2010, Keilani et al., 2012). Here, we investigated whether heterozygous deletion of *Hexb* exacerbated pathology and/or caused earlier onset of disease-related signs in *App*^*KI/KI*^ mice. While *Hexb* heterozygosity did not increase the abundance of its substrate, GM2 ganglioside, it did decrease the amount of GM1 ganglioside in the cerebral cortex. Further, even though *Hexb* heterozygosity did not induce substantial memory impairments in the Y-maze or the Novel Object Recognition test, it did cause a flexibility deficit in the Reversal Phase of the Morris water maze. *Hexb* heterozygosity also reduced amyloid burden in a regional manner in the brain and caused time-specific increases in the microglial marker IBA1.

Our results add to a complex literature on heterozygous deletion of lysosomal proteins. Cheng et al. (Cheng et al., 2017) showed that heterozygosity of another lysosomal hydrolase, cathepsin D, did not increase Aβ levels or deposition, or gross levels of APP, APP C-terminal fragments, GFAP or IBA1 in APPsw/PS1dE9 transgenic mice. In contrast, heterozygous deletion of *Npc1*, which codes for a lysosomal protein involved in cholesterol trafficking, was found to increase Aβ_42_ levels and amyloid plaque deposition in APP/PS1 transgenic mice (Erickson et al., 2008).

Genetic studies have also indicated that lysosomal network genes are an important factor in determining AD risk (Cruchaga et al., 2014, Gao et al., 2018, Lambert et al., 2013), and these genes predominantly lie along the endo-lysosomal pathway. Consistently, endocytic disruptions occur very early in the course of AD (Cataldo et al., 2000, Nixon, 2017). However, the lysosomal enzyme genes *HEXB, CTSD* (Beyer et al., 2005, Papassotiropoulos et al., 2000, Paz et al., 2015, Sierksma et al., 2020), and more recently *CTSH*, *CTSB*, and *IDUA* (Bellenguez et al., 2020)*,* also harbour associations with AD. As these loci each confer small increases in risk for disease, there is a need to evaluate more subtle measures of disease burden in *in vivo* studies that investigate single genes. Considering specific cell types and/or brain regions and employing more sensitive behavioural measures may reveal evidence of disruptions that would otherwise be unnoticed. The study evaluating the impact of cathepsin D haplodeficiency in an AD mouse (Cheng et al., 2017) focused on measurement of a few key AD-related proteins and did not rule out alterations in subtle behavioural measures or other neuropathological markers. Although our study did not reveal substantial memory impairments resulting from *Hexb* heterozygosity in the *App*^*KI/KI*^ mouse, it did demonstrate that *Hexb* is not entirely haplosufficient. *App*^*KI/KI*^; *Hexb*^*+/-*^ mice had impairments in the Reversal Phase of the Morris water maze at 42 to 44 weeks of age that were absent in *App*^*KI/KI*^; *Hexb*^*+/+*^ mice, suggesting deficits in behavioural flexibility. Future studies may show more severe phenotypes at later time points.

The *App*^*KI/KI*^; *Hexb*^*+/+*^ mice displayed reduced activity in the Open Field test at 40 to 41 weeks of age. Although two studies have reported no changes in activity in nine-month-old *App*^*KI/KI*^ mice (Latif-Hernandez et al., 2019, Mehla et al., 2019), they utilised a different testing duration and open field equipment with different dimensions. Gender of both the experimenter (Sorge et al., 2014) and mice (Nagy and Glaser, 1970, Tucker et al., 2016) is known to affect behavioural measures. Our study used a female experimenter to assess male mice, while male mice were assessed by a male experimenter in Mehla et al. (2019) and female mice were assessed by a female experimenter in the study by Latif-Hernandez et al. (2019); furthermore, Latif-Hernandez et al. (2019) used repeated behavioural measures. Importantly, the reduction in Open Field activity reported here was also observed in the Y-maze and is consistent with the phenotype reported in our previous study conducted under the same conditions (Whyte et al., 2018). The later age of onset here is potentially due to the different background strain of mice compared with our earlier study. We now demonstrate that this phenotype is reproduced by *Hexb* heterozygosity. Given that reduced activity was caused by both *Hexb* heterozygosity and *App* knock-in mutations and there was no additive effect when these mutations were combined, it is possible that the familial AD mutations in the *App*^*KI/KI*^ mice and *Hexb* heterozygosity affect activity via the same pathway.

It is unlikely that the hypoactivity in *App*^*KI/KI*^; *Hexb*^*+/+*^, *App*^*KI/KI*^; *Hexb*^*+/-*^ and *App*^*+/+*^*; Hexb*^*+/-*^ mice, or the impairment in behavioural flexibility observed in *App*^*KI/KI*^; *Hexb*^*+/-*^ mice are related to the amount of Aβ. *App*^*KI/KI*^ mice had severe amyloid pathology in the absence of substantial memory deficits. Furthermore, *Hexb* heterozygosity appeared to alter the trajectory of Aβ deposition such that there was less Aβ plaque in the orbital cortex and hippocampus of 46-week-old *App*^*KI/KI*^; *Hexb*^*+/-*^ mice compared with *App*^*KI/KI*^; *Hexb*^*+/+*^ mice, and a lower amount of Aβ_42_ in the hippocampus. This result was unexpected and reveals a complex role for the lysosomal system in plaque biology.

This complexity is also found in the literature, where the lysosomal system also has a complex role in the balance between plaque degradation and deposition. Reduced autophagy, mediated by heterozygous knock-out of *Becn1,* increases extracellular and intraneuronal Aβ in the hemizygous T41 transgenic AD mouse (Pickford et al., 2008). In contrast, Nilsson et al. (2013) dramatically reduced amyloid plaque deposition by using conditional knock-out of autophagy-related gene 7 (*Atg7*) in the APP23 AD mouse model.

The microglial lysosomal system also interacts with amyloid plaque burden in a complex way. Huang and colleagues (Huang et al., 2021) demonstrated that reducing lysosomal delivery of Aβ in microglia (by knocking out phagocytosis receptors) dramatically reduced dense core plaque burden, demonstrating amyloid plaques are at least in part constructed in the lysosomal system. Further, construction of dense amyloid plaques in this study was neuroprotective. The studies conducted by Nilsson et al*.*, Pickford et al*.*, and Huang et al. (Huang et al., 2021, Nilsson et al., 2013, Pickford et al., 2008) show that the lysosomal system is important for both degradation and construction of amyloid plaques. In the current study, microglial IBA1, TREM2, and CD68, and astrocytic GFAP staining were increased in *App* knock-in mice. Further, increases in microglial IBA1 staining at 32 weeks were followed by decreased amyloid plaque burden at 46 weeks in the hippocampus as a result of *Hexb* heterozygosity in the *App* NL-G-F background. Although we cannot prove this directly, perhaps the increase in numbers of microglia at 32 weeks is the cause of the decrease of amyloid plaque observed at 46 weeks in the *App*^*KI/KI*^*; Hexb*^*+/-*^ mice compared with the *App*^*KI/KI*^*; Hexb*^*+/+*^ mice. Curiously, changes in IBA1 staining were not accompanied by changes in pro-inflammatory cytokines. This is consistent with another study that showed a lack of robust cytokine increase (for IL-6 and IL-1β) in the brain when IBA1- and GFAP-positive staining had clearly already increased in response to amyloid plaques (Kaur et al., 2020). This same study did however note some increase in TNFα. Our current study adds to the scientific literature by demonstrating that partially reducing lysosomal function decreases amyloid plaque burden but (similar in theme to Huang et al., 2021) at the same time this results in worse functional outcomes for the brain.

GM1 ganglioside was also significantly reduced independently by both *App*^*KI/KI*^ and *Hexb*^*+/-*^ genotypes. Reduction of GM1 ganglioside has been observed before in Huntington's disease model mice where it was responsible for a reduction in pro-survival signalling through AKT/PKB (Maglione et al., 2010) and could represent a neurodegenerative state. *App*^*KI/KI*^ mice accumulated GM2 and GM3 gangliosides in the cerebral cortex. Interestingly, an increase in GM2 ganglioside happened at the same time as β-hexosaminidase activity was also increased. This increase in lysosomal enzyme activity is consistent with other studies that show increases of β-hexosaminidase in neurons from the brains of people who lived with AD (Cataldo et al., 1991). Collectively, this demonstrates lysosomal network dysfunction caused by mutations in the *App* gene. To our knowledge, this is the first report of elevated GM2 and GM3 in the *App*^*NL-G-F/NL-G-F*^ mouse model, and is consistent with GM2 and GM3 elevations in the cortex of both human AD (Kracun et al., 1992, Kracun et al., 1991, Molander-Melin et al., 2005), and the APP^SL^, APP^SL^/PS1^M146L^ and TgCRND8 mouse models (Barrier et al., 2007, Yang et al., 2014). It is noteworthy that *Hexb* heterozygosity did not further increase the amount of GM2 in *App*^*KI/KI*^ cortical homogenates. We cannot rule out very localised increases in GM2, such as an elevation in detergent-resistant membranes, which has been reported in the frontal cortex of AD patients (Molander-Melin et al., 2005), or in neuronal autolysosomes, the primary site of elevated GM2 in the TgCRND8 mouse (Yang et al., 2014). However, there was no overall change in GM2 with *Hexb* heterozygosity in an already stressed system (*App*^*KI/KI*^ background).

In conclusion, *Hexb* heterozygosity in *App*^*KI/KI*^ mice did not induce substantial memory impairments but it did lead to impairment of memory flexibility and activity in the Open Field test. These effects were observed at the same time as *Hexb* heterozygosity decreased amyloid plaque burden in the *App*^*KI/KI*^ brain. This demonstrated that the lysosomal enzyme gene, *Hexb*, is haploinsufficient in the context of AD. A growing body of literature suggests the lysosomal system has a complex relationship with amyloid plaques, being involved with both the destruction of Aβ, and the incorporation of Aβ into amyloid plaques, which is thought to protect the brain. Results from this study are consistent with this consensus.

## Ethics

The authors certify that formal approval to conduct the experiments described has been obtained from the animal subjects review board of SAHMRI and the University of Adelaide.

## Funding

This work was supported by an Australian Rotary Health/Rotary Club of Adelaide Funding Partner Scholarship and a Research Training Program Scholarship, awarded to LSW. The study was funded by Lysosomal Health in Ageing, Lifelong Health Theme, SAHMRI10.13039/100009843.

## CRediT authorship contribution statement

**Lauren S. Whyte**: Conceptualization, Methodology, Formal analysis, Writing – original draft, Writing – review & editing. **Celia Fourrier**: Methodology, Formal analysis, Writing – review & editing. **Sofia Hassiotis**: Methodology, Formal analysis, Writing – review & editing. **Adeline A. Lau**: Conceptualization, Formal analysis, Writing – review & editing. **Paul J. Trim**: Methodology, Writing – review & editing. **Leanne K. Hein**: Methodology, Writing – review & editing. **Kathryn J. Hattersley**: Methodology, Writing – review & editing. **Julien Bensalem**: Formal analysis, Writing – review & editing. **John J. Hopwood**: Conceptualization, Writing – review & editing. **Kim M. Hemsley**: Conceptualization, Formal analysis, Writing – review & editing. **Timothy J. Sargeant**: Conceptualization, Project administration, Formal analysis, Writing – original draft, Writing – review & editing.
