# Supplementary material for: Lysosomal gene Hexb displays haploinsufficiency in a knock-in mouse model of Alzheimer’s disease
Source: IBRO Neurosci Rep. 2022 Jan 20;12:131–41. doi: 10.1016/j.ibneur.2022.01.004 (PMC8819126; doi:10.1016/j.ibneur.2022.01.004)
Supplement: Supplementary file 1 — Supplementary material [file mmc1.docx]

**Supplementary Materials and Methods**

**Materials**

The Y-maze and Morris water maze were manufactured by Acrillix Plastics Pty Ltd (Adelaide, Australia). EthoVision XT10 software was purchased from Noldus (Wageningen, The Netherlands). Haymes, white ceiling paint (CWE10) was purchased from Inspirations Paint (Adelaide, Australia). Heat Mats (N2867, AC220-240V/50Hz 20W) were from Reptile One (Ingleburn, NSW, Australia). Cathepsins D/E fluorogenic substrate (219360) was from Calbiochem (California, United States), 4-Methylumbelliferyl-β-ᴅ-galactoside (M1095) was from Melford (Suffolk, United Kingdom), 4-Methylumbelliferyl-*N*-acetyl-β-ᴅ-glucosaminide (M-2133) and Z-Phe-Arg 7-amido-4-methylcoumarin hydrochloride (C9521) were from Sigma (Missouri, United States).

Anti-human amyloid β (N) 82E1 mouse IgG (10323) was from IBL America (Minneapolis, MN, United States). Anti-CD68 (ab125212), anti-LAMP1 (ab24170) and anti-Synaptophysin (ab32127) rabbit antibodies were from Abcam (Cambridge, UK). Anti-GFAP rabbit polyclonal antibody (Z0334) was from DAKO (Glostrup, Denmark). Anti-IBA1 rabbit antibody (019-19741) was from Wako (Osaka, Japan). Anti-TREM2 sheep polyclonal antibody (AF1729) was from R&D Systems (Minneapolis, MN, United States).

Secondary antibodies biotin donkey α mouse IgG (H+L) (715-065-150), biotin donkey α rabbit (711-065-152) and biotin donkey α sheep IgG (H+L) (713-065-147) along with normal donkey serum (017-000-121) and Peroxidase-conjugated Streptavidin (016-030-084)) were purchased from Jackson ImmunoResearch Laboratories (West Grove, PA, United States). Vectastain Elite ABC Kit (PK-6100) was purchased from Vector Laboratories. DAB+ substrate chromogen system (K3468) was from DAKO.

cOmplete, EDTA-free protease inhibitor cocktail (04693132001) was purchased from Sigma. VibraCell ultrasonic processor was from Sonics and Materials (Newtown, CT, USA). Human/rat β amyloid (40) ELISA kit II (294-64701) and human/rat β amyloid (42) ELISA kit, high-sensitive (292-64501) were purchased from Wako. Synthetic peptides [Gly22]-amyloid beta 1-42 Arctic human (SCP0030), [Gly22]-amyloid beta 1-40 Arctic human (SCP0029), amyloid beta 1-42 rat (SCP0038) and amyloid beta 1-40 rat (SCP0037) were purchased from Sigma. Mouse IL-1 beta (BMS6002), Mouse IL-6 (BMS603-2) and Mouse TNF alpha (BMS607) ELISA kits were purchased from ThermoFisher Scientific. Total protein was determined using a Micro-BCA Protein Assay Kit (23225) from Thermo Scientific.

Internal standards GM1 (d18:1/18:0) {N-*omega*-CD_3_-octadecanoyl monosialoganglioside GM_1_ (NH_4_^+^ salt) (2050), GM2 (d18:1/18:0) {N-*omega*-CD_3_-octadecanoyl monosialoganglioside GM_2_ (NH_4_^+^ salt) (2051) and GM3 (d18:1/18:0) {N-*omega*-CD_3_-octadecanoyl monosialoganglioside GM_3_ (NH_4_^+^ salt)} (2052) were purchased from Matreya LLC (Pleasant Gap, PA, United States).

**Animals**

Mice were group-housed from weaning in individually ventilated cages containing sterile Eco-Pure Aspen chips as bedding (Datesand, UK), in a specific and opportunistic pathogen-free laboratory. Cages were maintained between 18–24°C and 35-75% humidity with a 12-h light: 12-h dark cycle. Mice were weighed fortnightly from four weeks of age and housed individually nine days prior to behavioural testing.

**Behaviour**

Mice were housed behind a curtain in the behavioural room from the day before testing commenced. Testing was conducted in the lights-on phase between the hours of 0700 and 1600, in a randomised order. Testing on each mouse was carried out in the same order each day. All sessions were recorded using video tracking software (EthoVision XT10).

*Y-maze*

Mice were placed in the centre of the Y-maze and allowed to explore freely for 5 min. The number of arm entries was manually recorded. EthoVision XT10 software was used to determine total path length and average speed. Percentage alternation was calculated as the number of alternations divided by the number of possible alternations multiplied by 100 (where the number of possible alternations was equal to the total number of arm entries minus 2). The Y-maze was cleaned with 70% ethanol between successive mice.

*Open field*

Mice were placed in a 45 × 45× 35 (high) cm white corflute box and allowed to explore freely for 5 min. EthoVision XT10 software was used to divide the floor into 16 (4 × 4) zones measuring 11.25 × 11.25 cm. The number of zone entries, total path length and average speed was determined using EthoVision. The number of rears made up onto hindlimbs and time spent grooming was scored manually using the manual scoring feature of EthoVision. The arena was cleaned with 70% ethanol between successive mice.

*Novel object recognition test*

Mice were exposed to two identical objects (either two Duplo block towers or two tissue culture flasks filled with sand) for 10 min on the day following the open field test. On the third day, mice were re-introduced to the arena under identical conditions, except with one block tower and one sand-filled flask. The positions of the novel and familiar objects were counter-balanced between mice to avoid innate preference for a location. The time spent interacting with each object was recorded, with interaction scored whenever the mouse sniffed or touched the object while looking at it (when the distance between the nose and the object was less than 2 cm). Climbing onto an object (in the absence of sniffing) or chewing the object was not counted as exploration [1]. The percentage of total interaction time spent with the novel object and familiar object calculated. The objects and arena were cleaned with 70% ethanol between successive mice.

*Morris water maze*

A white, featureless high-density polyethylene pool (1200 mm diameter and 400 mm high) was filled with water to approximately 30 cm deep, made opaque by the addition of nontoxic white paint and maintained between 20-23°C. The pool was arbitrarily divided into four quadrants: north west (NW), north east (NE), south west (SW) and south east (SE), and a 10 cm diameter platform was positioned 35 cm from the wall in the NW quadrant 1 cm below the water level. Visual cues (monochromatic geometric shapes) were presented on the W and S walls. The experimenter and mice were along the E wall behind a curtain to obscure the pool and visual cues during the test. During the five-day Acquisition Phase, mice underwent four swims/day beginning in each of the four quadrants. The time taken to find the platform was recorded using EthoVision software. Mice that did not find the platform within 90 s were guided to the platform and allowed to remain there for approximately 10 s prior to removal from the pool. The swim time for each of the four swims was averaged to give a swim time for each of the five days for each mouse. An inter-trial interval of at least 10 min was maintained between successive swims, during which mice were returned to their home cages, which were warmed using heat mats, and provided with fresh tissues. A probe test was conducted on day 6, during which the platform was removed from the pool and each mouse given a single 90 s swim, starting from the SE quadrant. EthoVision software was used to record the time spent in each of the four quadrants. A five-day Reversal Phase commenced on the day following the probe test, and this was followed by a reversal probe and visual test 24 h after the final day of the Reversal Phase. During the visual test, visual cues were removed from the walls and the visible platform (colourful flag on platform, sitting just below water’s surface) placed 35 cm from the wall in the SW quadrant. Mice were given up to four swims in opaque water, from each of the four quadrants, or until such time as a latency of <10 s was achieved.

**Measurement of lysosomal enzyme activities**

Briefly, cathepsins D/E activity, β-galactosidase and β-hexosaminidase activity were measured using 7 μg of protein with 200 μM 7-Methoxycoumarin-4-yl acetyl-Gly-Lys-Pro-Ile-Leu-Phe~Phe-Arg-Leu-Lys(Dnp)-D-Arg-NH₂ [2], 500 μM 4-Methylumbelliferyl-β-ᴅ-galactoside [3] and 1.08 mM 4-Methylumbelliferyl-*N*-acetyl-β-ᴅ-glucosaminide [4] respectively. Cathepsins B/L activity was measured using 4 μg protein with 50 μM Z-Phe-Arg 7-amido-4-methylcoumarin hydrochloride [5].

**Image analysis**

For image analysis, the cortex was coronally divided into three equal regions, which were designated as “rostral”, “intermediate” and “caudal”. In addition, an area of 0.4 mm^2^ enriched for orbital cortex was examined as well as the hippocampus, (which for the purpose of this study included all fields of the hippocampus proper present in sections, predominately CA1 and CA2, with some CA3, in addition to the dentate gyrus, hippocampal fissure and subiculum), thalamus and inferior colliculus. Brain regions were defined with reference to anatomical landmarks using a mouse brain atlas [6].

**Aβ ELISAs**

For cortical homogenates, TBS-soluble material was separated from insoluble material in guanidine-HCl as described previously [7]. Briefly, homogenates were centrifuged at 186,000 g for 20 min at 4°C. The supernatant was combined with 6 M guanidine/50 mM Tris-HCl pH 7.4 to give a final concentration of 0.5 M guanidine and designated as the TBS-soluble fraction. The pellet was washed with 50 mM TBS and centrifuged at 186,000 g for 5 min at 4°C. After discarding the supernatant, the pellet was resuspended in 6 M guanidine/50 mM Tris-HCl pH 7.4 with cOmplete, EDTA-free protease inhibitor cocktail and 1 mM EDTA (7.5x volume of starting material) and solubilised by sonicating with a VibraCell ultrasonic processor for 30 s. Samples were centrifuged at 186,000 g for 5 min at 25°C and the supernatant diluted 12 times with 50 mM TBS in order to reduce the concentration of guanidine to 0.5 M. Samples were centrifuged at 21,130 g for 5 min at 4°C to remove debris and the supernatant used as the GuHCl fraction.

**IL-6, TNF-α, and IL-1β ELISAs**

Right cortex samples were homogenised in lysis buffer containing 20 mM Tris HCl, 150 mM NaCl, 1 mM Na_2_EDTA, 1 mM EGTA, 1% Triton, 2.5 mM sodium pyrophosphate, 1 mM β-glycerophosphate, 1 mM Na_3_VO_4_ and Roche cOmplete EDTA-free protease inhibitor cocktail (Sigma Aldrich) and were sonicated at 25 Hz for 30 seconds.

IL-1β, IL-6 and TNF-α were measured following manufacturer’s instructions. All samples were run in duplicate. The analytical sensitivity was 1.2 pg/mL for IL-1β, 6.5 pg/mL for IL-6 and 3.7 pg/mL for TNF-alpha; the assay range was 7.8-500 pg/mL for IL-1β and 31.3-2,000 pg/mL for IL-6 and TNF-α; the interassay coefficient of variation was 8.9% for IL-6 and 5.7% for IL-1β and TNF-α and the intraassay coefficient of variation was 4.7% for IL-1β, 5% for IL-6 and 6.5% for TNF-α. The Victor3™ Plate Reader (PerkinElmer) was used for colorimetric reading. For each sample, the final concentration was calculated after adjusting for the total concentration of protein loaded.

**Supplementary References**

1. Leger, M., Quiedeville, A., Bouet, V., Haelewyn, B., Boulouard, M., Schumann-Bard, P. & Freret, T. (2013) Object recognition test in mice, *Nat Protoc.* **8**, 2531-7.

2. Yasuda, Y., Kageyama, T., Akamine, A., Shibata, M., Kominami, E., Uchiyama, Y. & Yamamoto, K. (1999) Characterization of new fluorogenic substrates for the rapid and sensitive assay of cathepsin E and cathepsin D, *Journal of biochemistry.* **125**, 1137-43.

3. Ho, M. W. & O'Brien, J. S. (1971) Differential effect of chloride ions on -galactosidase isoenzymes: a method for separate assay, *Clin Chim Acta.* **32**, 443-50.

4. Leaback, D. H. & Walker, P. G. (1961) Studies on glucosaminidase. 4. The fluorimetric assay of N-acetyl-beta-glucosaminidase, *Biochem J.* **78**, 151-6.

5. Sargeant, T. J., Lloyd-Lewis, B., Resemann, H. K., Ramos-Montoya, A., Skepper, J. & Watson, C. J. (2014) Stat3 controls cell death during mammary gland involution by regulating uptake of milk fat globules and lysosomal membrane permeabilization, *Nat Cell Biol.* **16**, 1057-68.

6. Paxinos, G. & Franklin, K. B. J. (2001) The Mouse Brain in Stereotaxic Coordinates in *2nd edition*, Academic Press, San Diego, CA.

7. Iwata, N., Mizukami, H., Shirotani, K., Takaki, Y., Muramatsu, S., Lu, B., Gerard, N. P., Gerard, C., Ozawa, K. & Saido, T. C. (2004) Presynaptic localization of neprilysin contributes to efficient clearance of amyloid-beta peptide in mouse brain, *J Neurosci.* **24**, 991-8.
