# Supplementary material for: Lysosomal gene Hexb displays haploinsufficiency in a knock-in mouse model of Alzheimer’s disease
Source: IBRO Neurosci Rep. 2022 Jan 20;12:131–41. doi: 10.1016/j.ibneur.2022.01.004 (PMC8819126; doi:10.1016/j.ibneur.2022.01.004)
Supplement: Supplementary file 2 — Supplementary material [file mmc2.pdf]

Supplementary Figure 1.

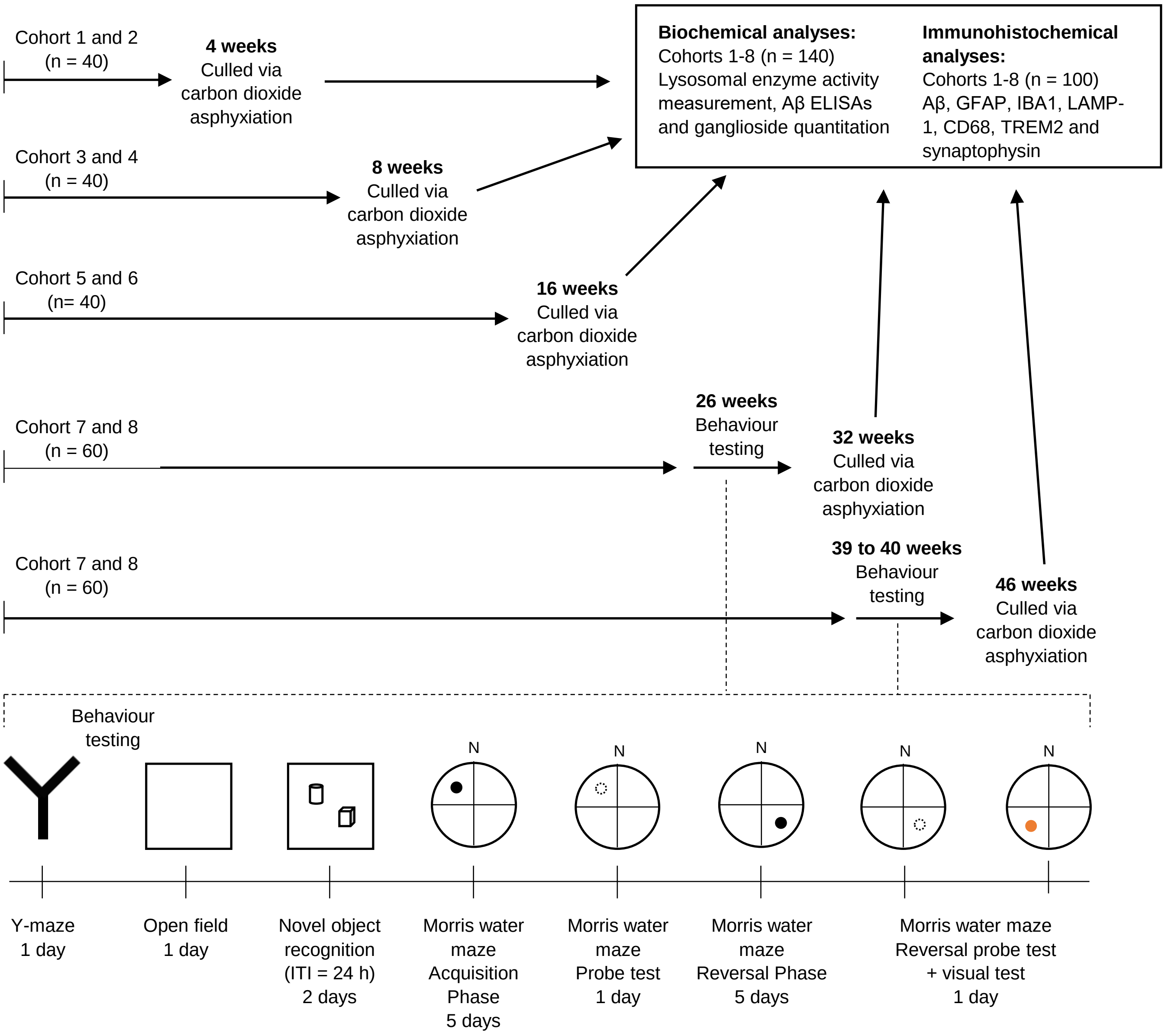

Supplementary Figure 1. Animal usage. Total numbers of mice used in each analysis is shown (ITI: inter-trial interval).

Supplementary Figure 2.

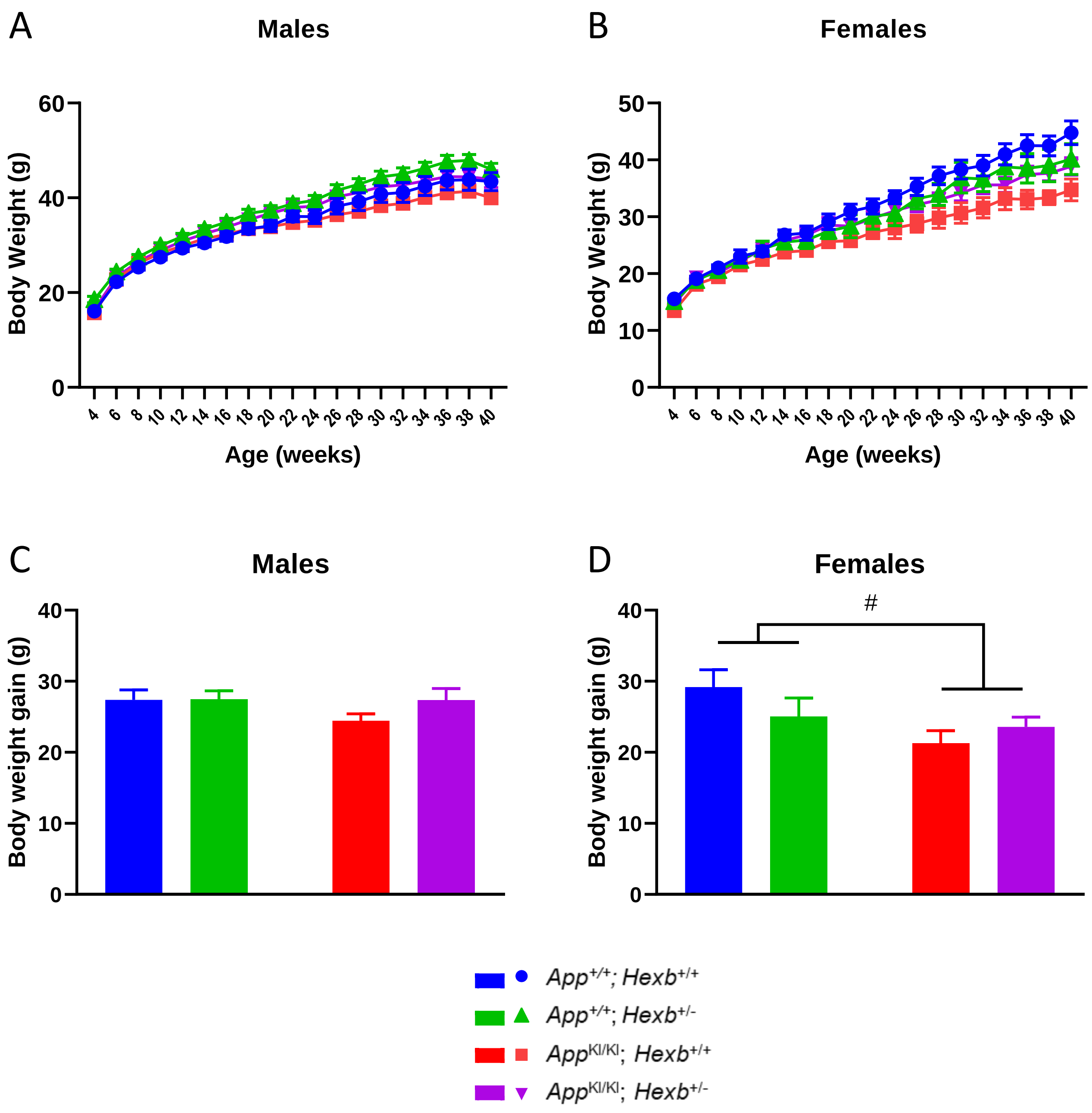

Supplementary Figure 2. Body weights. Average total body weight of *App*$^{+/+}$; *Hexb*$^{+/+}$, *App*$^{+/+}$; *Hexb*$^{+/-}$, *App*$^{KI/KI}$; *Hexb*$^{+/+}$ and *App*$^{KI/KI}$; *Hexb*$^{+/-}$ mice up to 40 weeks n = 15-21 (males, A), n = 5-10 (females, B), error bars = ± SEM. Total body weight gain between 4 weeks and 40 weeks of age. n = 15-16 (males, C), n = 5-8 (females, D); # p<0.05, *App* genotype effect (two-way ANOVA).

Supplementary Figure 3.

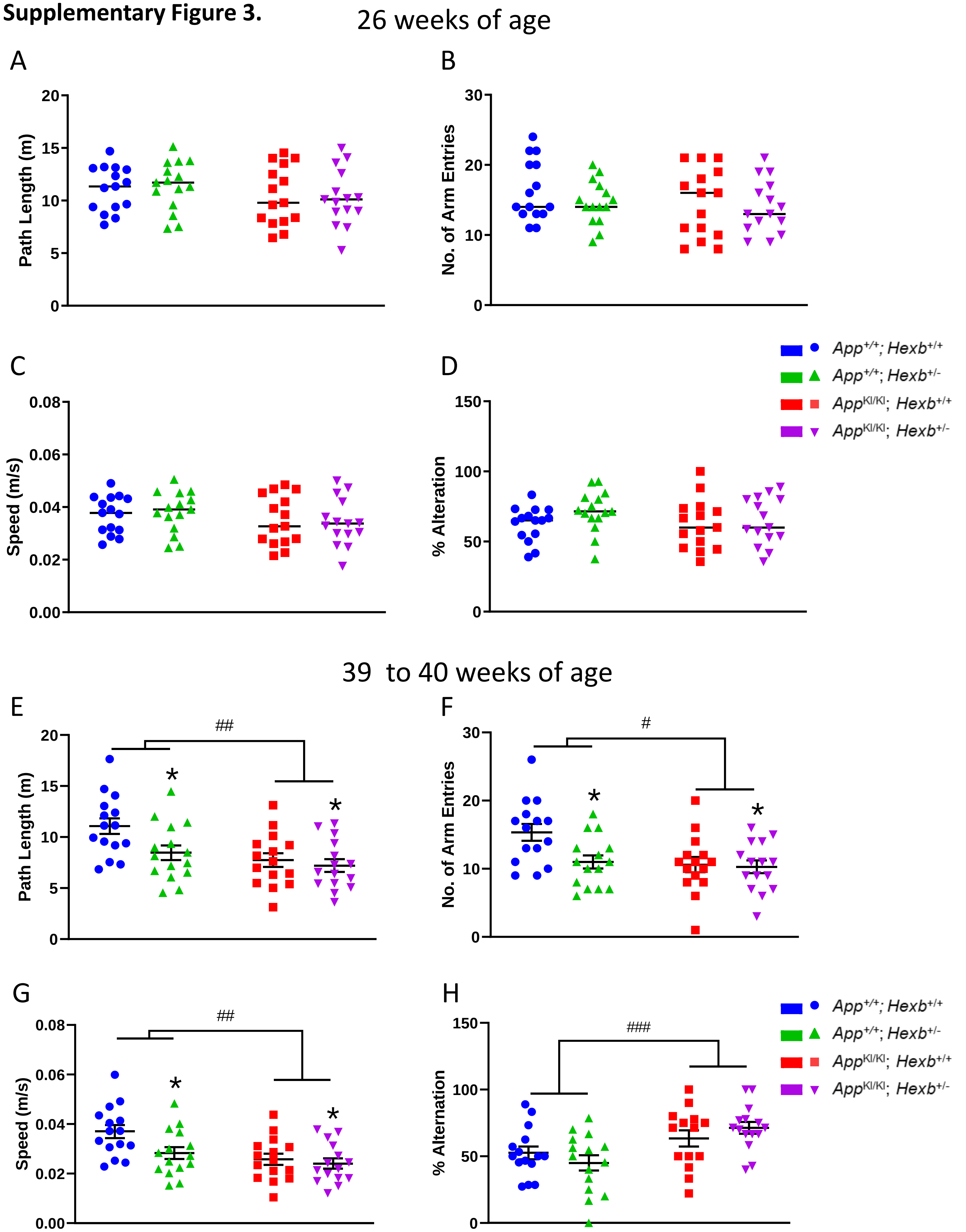

Supplementary Figure 3. Working memory and activity levels in the Y-maze at 26 and 39 weeks of age. Y-maze tests were conducted with male mice. During the 5-minute trial, path length travelled was recorded with EthoVision XT10 software (A, E), the number of arm entries was manually recorded (B, F) and the average speed was automatically recorded (C, G). Percent alteration was calculated as the number of alterations divided by the number of possible alterations multiplied by 100 (D, H). n= 14-15 male mice/group, error bars = ± SEM, * p<0.05, *Hexb* genotype effect; # p<0.05, ## p<0.01, ### p<0.001, *App* genotype effect (two-way ANOVA).

Supplementary Figure 4.

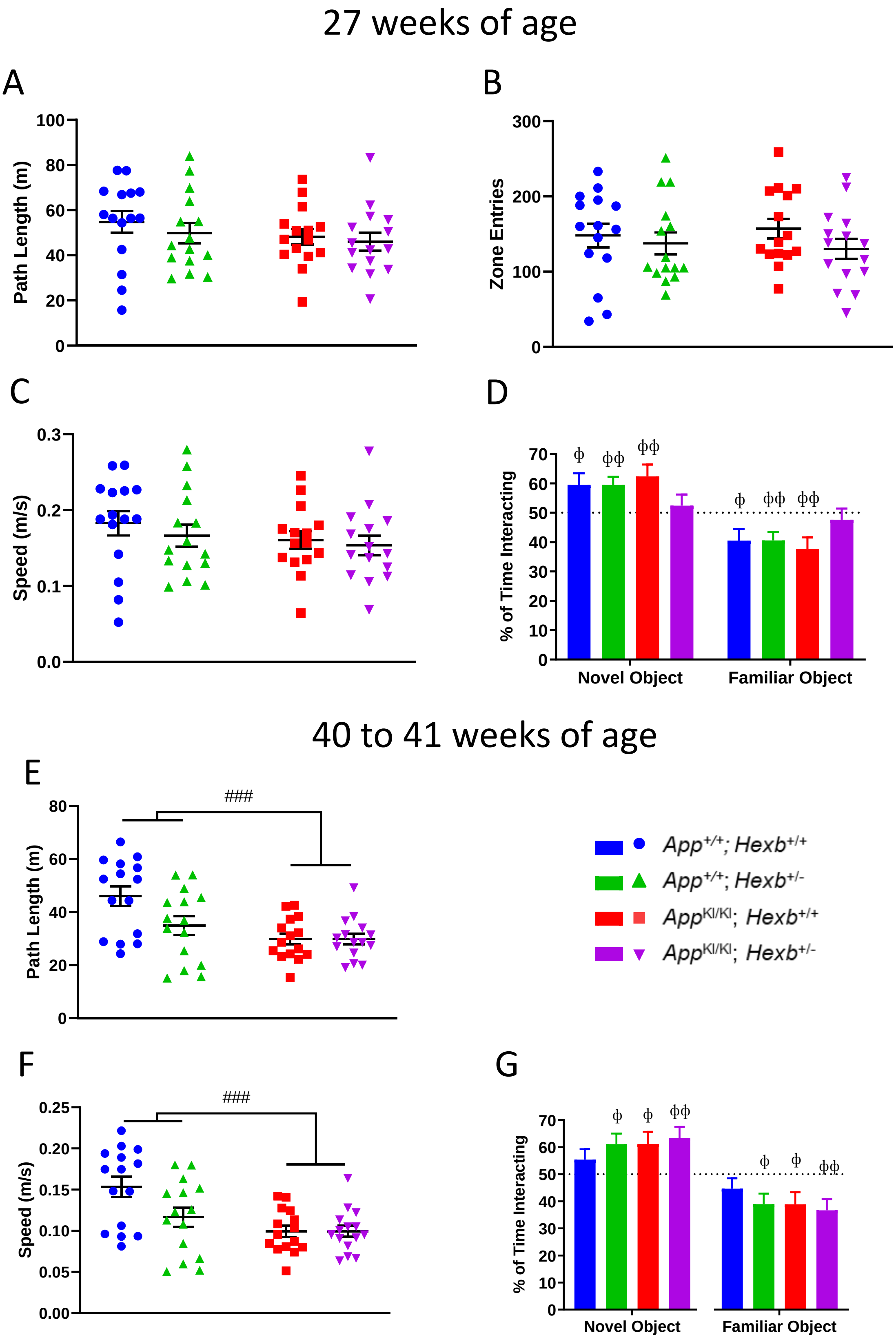

Supplementary Figure 4. Open Field activity and recognition memory in the Novel Object Recognition Test at 27 (A-D) and 40 to 41 (E-G) weeks. Locomotor and exploratory activity was assessed in an Open Field Test with male mice. Path length (A,E), zone entries (B) and average speed (C,F) were recorded. Recognition memory was assessed with the Novel Object Recognition Test. The percentage of interaction time with the novel and familiar object was calculated (D,G). n = 12-15 male mice/group for 26 weeks, and n = 9-15 male mice/group for 40-41 weeks. Error bars = ± SEM, (two-way ANOVA; A-C, E-F). ### $p<0.001$, *App* genotype effect (E-F: two-way ANOVA). ϕ $p<0.05$, ϕϕ $p<0.01$ compared with chance level (50 %) (analysed with one sample t test and two-way ANOVA, D,G).

Supplementary Figure 5.

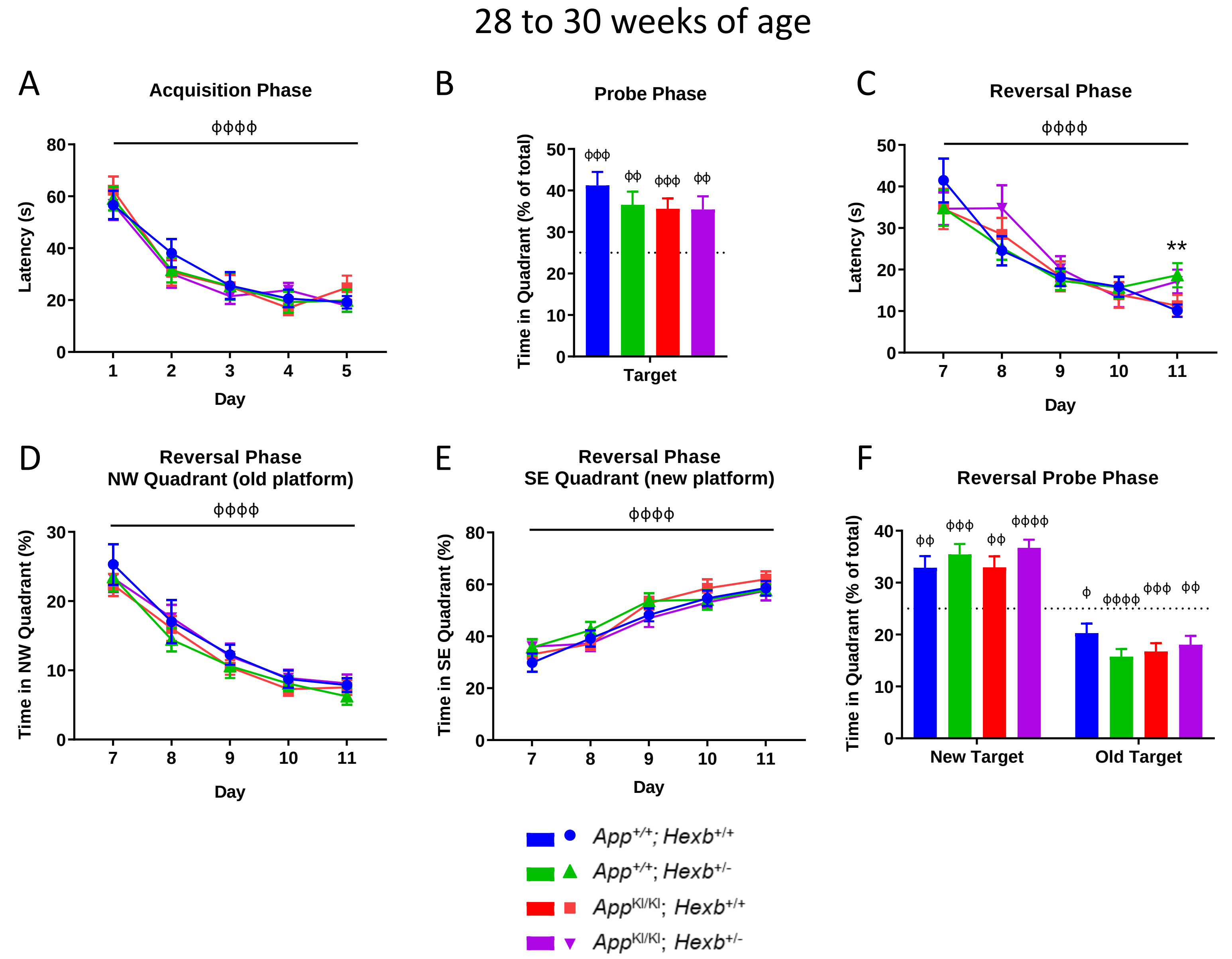

Supplementary Figure 5. Spatial memory and learning in the Morris Water Maze at 28 to 30 weeks. Male mice were evaluated in the Morris water maze. Average time to reach the platform in the Acquisition Phase is shown (A). In the Probe Phase, the platform was removed and mice were tested in their ability to recall its location. The percentage of time spent in the target quadrant (B; dashed line = chance level). The platform was placed in the opposite quadrant of the pool for the duration of the Reversal Phase. Average time to reach the platform (C) and the percentage of swim time spent in the NW (old platform) (D) and SW (New platform) (E) quadrants was recorded. On Day 12 the platform was again removed from the pool and the percentage of time spent in the new target quadrant and the old target quadrant was measured (F; dashed line = chance level). n = 15 male mice/group, error bars = ± SEM. A; C-F: ϕϕϕϕ $p<0.0001$ time effect; ** $p<0.01$, *Hexb* genotype effect (2-step analysis: time, *App* genotype and Hexb genotype effects and time**App***Hexb* interaction analysed with a three-way repeated measures ANOVA; then individual time points analysed with a two-way ANOVA). B; F: ϕϕ $p<0.01$, ϕϕϕ $p<0.001$ compared with chance level (*i.e.* 25 %) (analysed with one sample t test and two-way ANOVA).

Supplementary Figure 6.

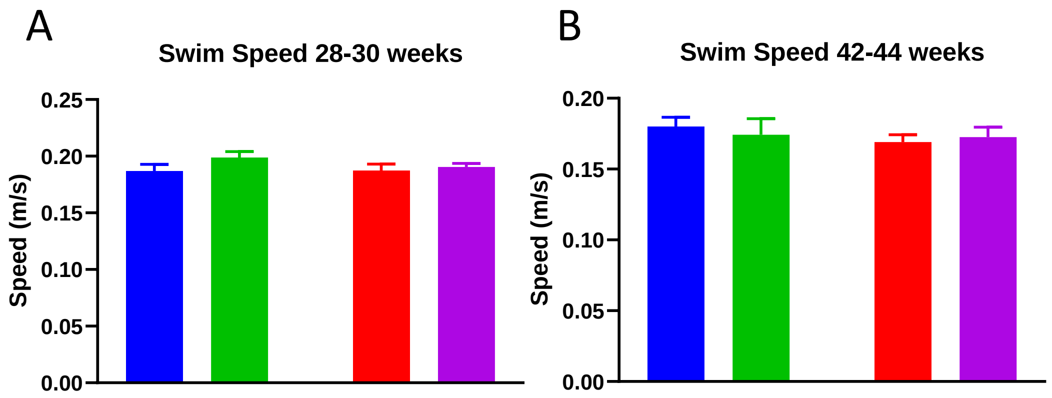

Supplementary Figure 6. Swim speed for Morris Water Maze. Average swim speed was recorded during the Probe Phases (A and B, two-way ANOVA). n = 15 male mice/group, error bars = ± SEM.

Supplementary Figure 7.

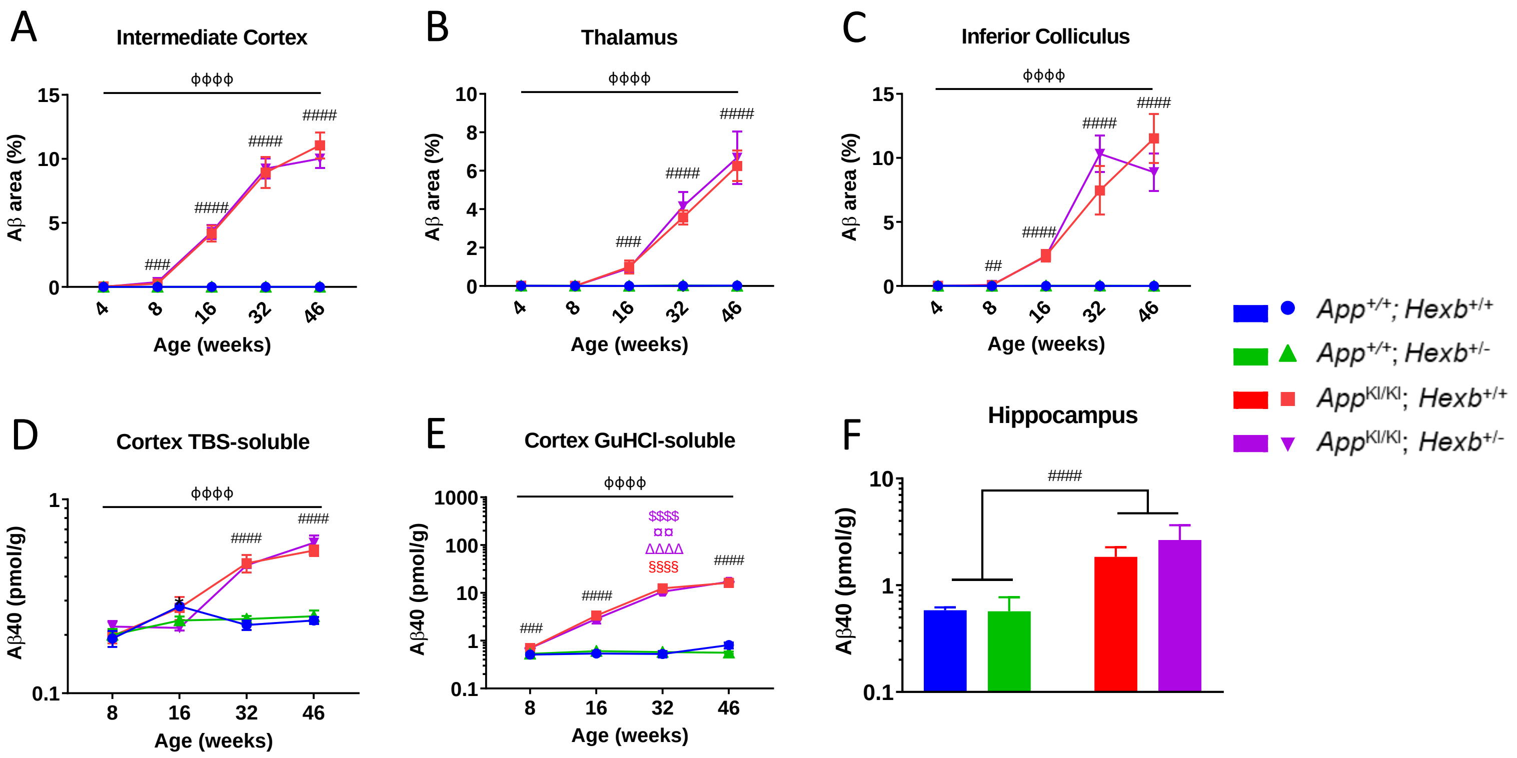

Supplementary Figure 7. Quantitation of histochemical staining and ELISA analysis of plaque burden related to Fig. 3. Quantification of the area of Aβ staining (stained with 82E1) in *App*$^{+/+}$; *Hexb*$^{+/+}$, *App*$^{+/+}$; *Hexb*$^{+/-}$, *App*$^{KI/KI}$; *Hexb*$^{+/+}$ and *App*$^{KI/KI}$; *Hexb*$^{+/-}$ mice at 4, 8, 16, 32 and 46 weeks was performed in the intermediate cortex, thalamus and inferior colliculus (A-C, n = 5 mice/group, error bars = ± SEM). Aβ40 peptide was quantified by ELISA in TBS-soluble (D) and GuHCl (E) fractions of cortical homogenates from 8-, 16-, 32- and 46-week-old mice and hippocampal homogenates from 46-week-old mice (F), n = 3-9 mice of mixed sex/group, error bars = ± SEM. A-E: ϕϕϕϕ p<0.0001 age effect; ## p<0.01, ### p<0.001, #### p<0.0001, *App* genotype effect; §§§§ p<0.0001 colour-indicated group vs *App*$^{+/+}$; *Hexb*$^{+/+}$; ¤¤ p<0.01 colour-indicated group vs *App*$^{KI/KI}$; *Hexb*$^{+/+}$; ΔΔΔΔ p<0.0001 colour-indicated group vs *App*$^{+/+}$; *Hexb*$^{+/-}$ (A-E, 2-step analysis: age, *App* genotype and *Hexb* genotype effects and age**App***Hexb* interaction analysed with a three-way ANOVA; then individual time points analysed with a two-way ANOVA followed by Tukey's multiple comparison tests when *App***Hexb* interaction p<0.05; F: two-way ANOVA).

Supplementary Figure 8.

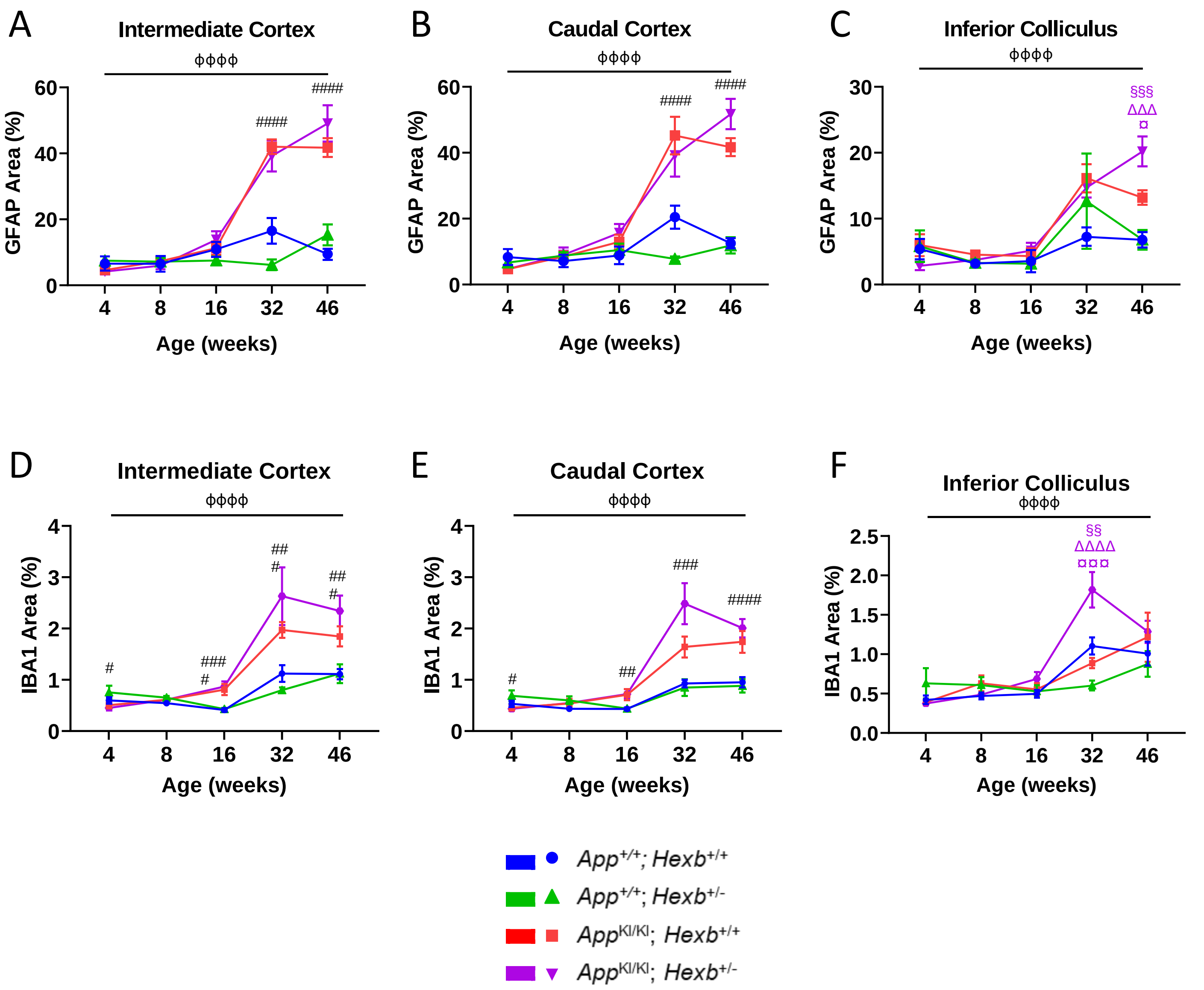

Supplementary Figure 8. Glial fibrillary acidic protein (GFAP) and ionised calcium binding adaptor molecule 1 (Iba1) staining related to Fig. 4. Quantification of the area of GFAP and Iba1 staining in *App*$^{+/+}$; *Hexb*$^{+/+}$, *App*$^{+/+}$; *Hexb*$^{+/-}$, *App*$^{KI/KI}$; *Hexb*$^{+/+}$ and *App*$^{KI/KI}$; *Hexb*$^{+/-}$ mice at 4, 8, 16, 32, and 46 weeks was performed in the intermediate cortex (A, D), caudal cortex (B, E) and inferior colliculus (C, F). n = 5 mice of mixed sex/group, error bars = ± SEM, ϕϕϕϕ p<0.0001 age effect; # p<0.05, ## p<0.01, ### p<0.001, #### p<0.0001, *App* genotype effect; §§ p<0.01, §§§ p<0.001 colour-indicated group vs *App*$^{+/+}$; *Hexb*$^{+/+}$; ¤ p<0.05, ¤¤¤ p<0.001 colour-indicated group vs *App*$^{KI/KI}$; *Hexb*$^{+/+}$; ΔΔ p<0.001, ΔΔΔΔ p<0.0001 colour-indicated group vs *App*$^{+/+}$; *Hexb*$^{+/-}$ (2-step analysis: age, *App* genotype and *Hexb* genotype effects and age**App***Hexb* interaction analysed with a three-way ANOVA; then individual time points analysed with a two-way ANOVA followed by Tukey's multiple comparison tests when *App***Hexb* interaction p<0.05).

Supplementary Figure 9.

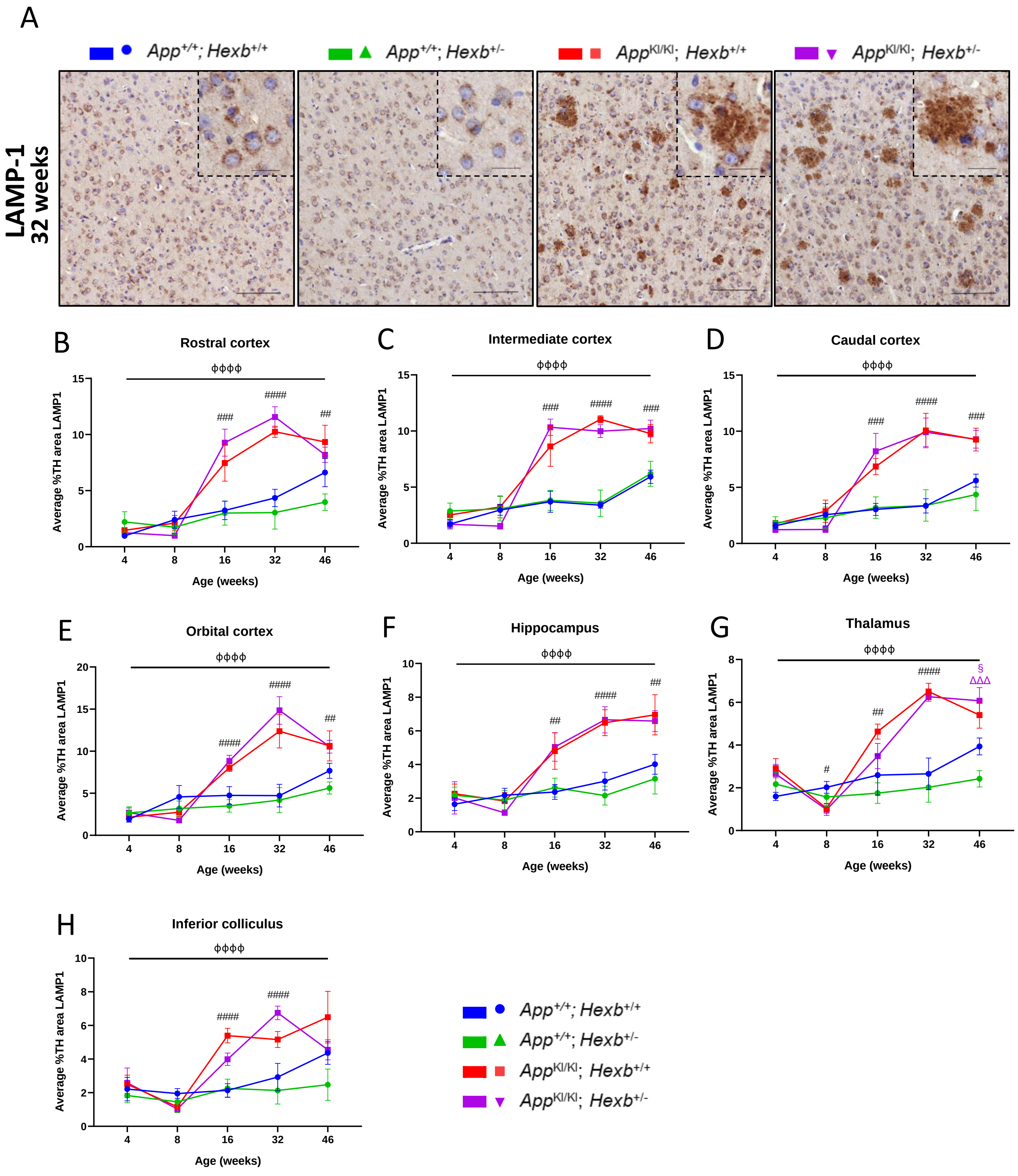

Supplementary Figure 9. Lysosomal-associated membrane protein 1 (LAMP-1) staining. Staining for LAMP-1 is shown in rostral cortex (A) Scale bar = 100 µm, inset scale bar = 20 µm. Quantification of the area of LAMP-1 staining in *App*$^{+/+}$; *Hexb*$^{+/+}$, *App*$^{+/+}$; *Hexb*$^{+/-}$, *App*$^{KI/KI}$; *Hexb*$^{+/+}$ and *App*$^{KI/KI}$; *Hexb*$^{+/-}$mice at 4, 8, 16, 32, and 46 weeks was performed in the rostral cortex (B), intermediate cortex (C), caudal cortex (D), orbital cortex (E), hippocampus (F), thalamus (G) and inferior colliculus (H). n = 5 mice of mixed sex/group, error bars = ± SEM, ϕϕϕϕ $p<0.0001$ age effect; ## $p<0.01$, ### $p<0.001$, #### $p<0.0001$, *App* genotype effect; § $p<0.05$ colour-indicated group vs *App*$^{+/+}$; *Hexb*$^{+/+}$; ΔΔΔ $p<0.001$ colour-indicated group vs *App*$^{+/+}$; *Hexb*$^{+/-}$ (2-step analysis: age, APP genotype and Hexb genotype effects and age**App***Hexb* interaction analysed with a three-way ANOVA; then individual time points analysed with a two-way ANOVA followed by Tukey's multiple comparison tests when *App***Hexb* interaction $p<0.05$).

Supplementary Figure 10.

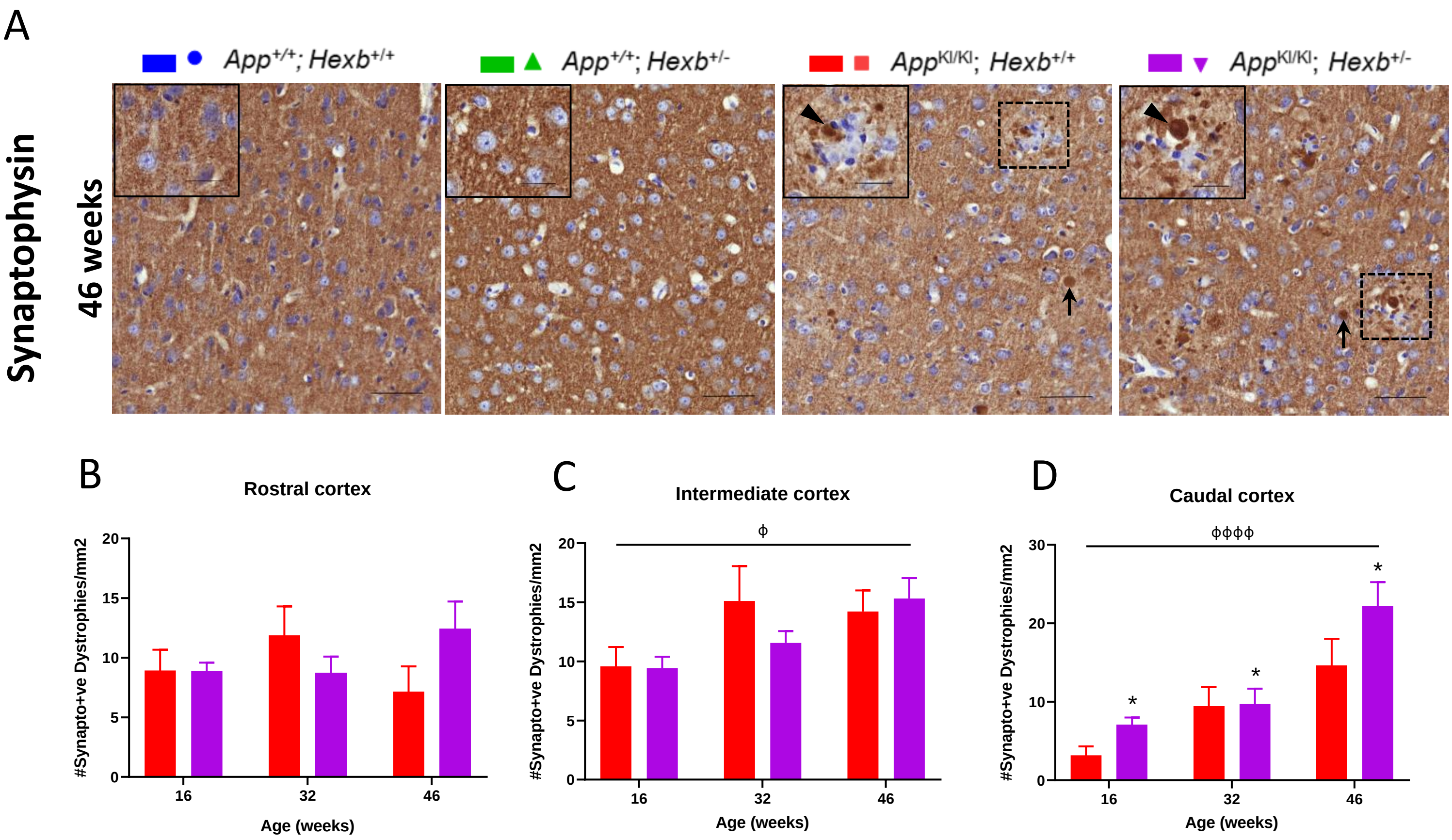

Supplementary Figure 10. Synaptophysin staining shows axonal dystrophies in *App* knock-in mice. Rostral cortex that was stained for synaptophysin is shown in panel A. Black arrowheads show swollen axonal dystrophies in enlarged boxes. Black arrows show individual axonal spheroids in neuropil. Scale bar = 50 µm, inset scale bar = 20 µm. Quantification of the number of synaptophysin-positive dystrophies/mm^2 in *App*$^{KI/KI}$; *Hexb*$^{+/+}$ and *App*$^{KI/KI}$; *Hexb*$^{+/-}$ mice at 16, 32 and 46 weeks was performed in the rostral cortex (B), intermediate cortex (C) and caudal cortex (D). n = 5 mice of mixed sex/group, error bars = ± SEM, ϕ $p<0.05$, ϕϕϕϕ $p<0.0001$ age effect; * $p<0.05$ *Hexb* genotype effect (two-way ANOVA).

Supplementary Figure 11.

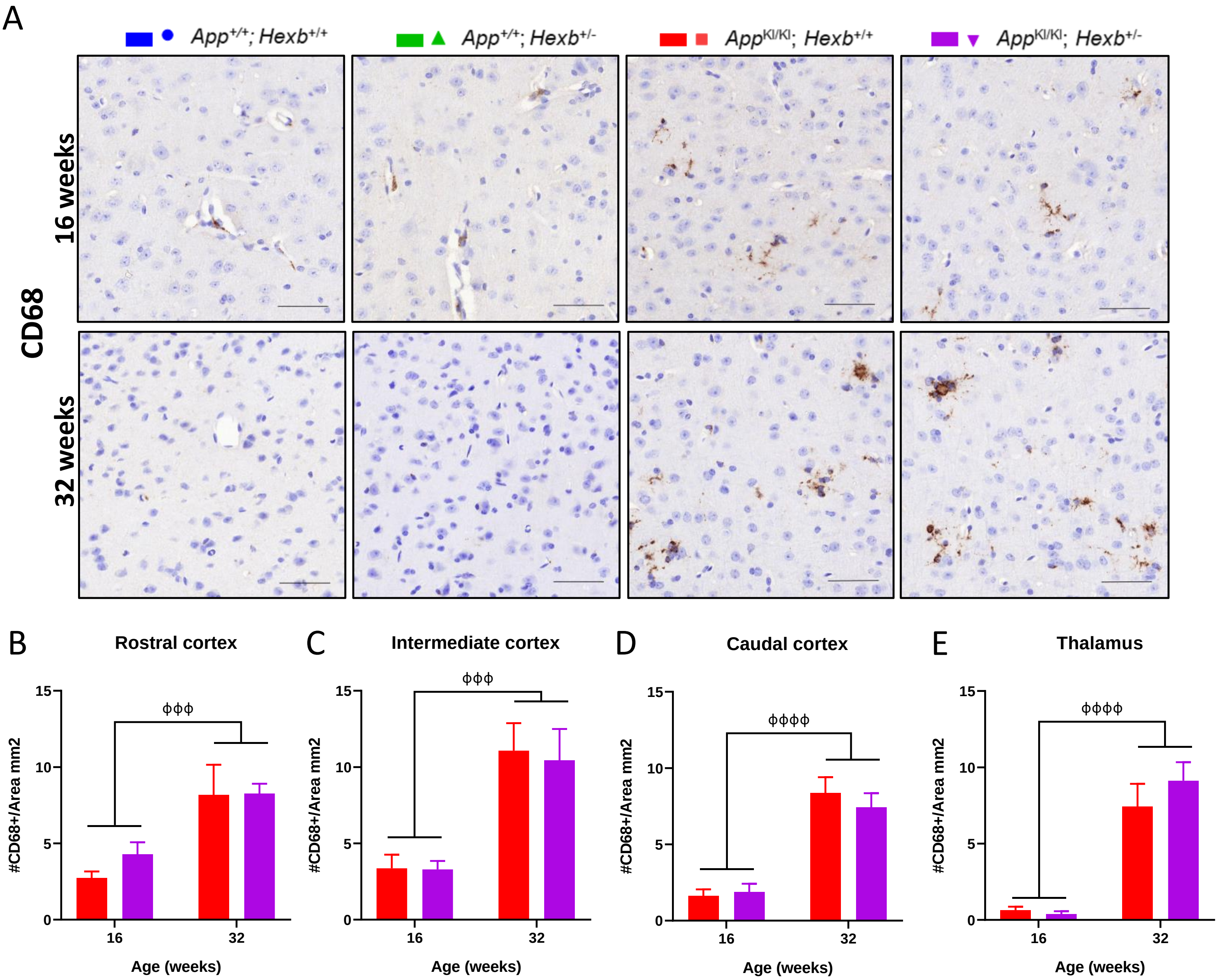

Supplementary Figure 11. Cluster of differentiation 68 (CD68) staining. Staining for CD68 is shown in rostral cortex (A). Scale bars = 50 µm. Quantification of the area of CD68 staining in *App$^{KI/KI}$*; *Hexb$^{+/+}$* and *App$^{KI/KI}$*; *Hexb$^{+/-}$* mice at 16 and 32 weeks was performed in the rostral cortex (B), intermediate cortex (C), caudal cortex (D), and thalamus (E). n = 5 mice of mixed sex/group, error bars = ± SEM, ϕϕϕ p<0.001, ϕϕϕϕ p<0.0001 age effect (two-way ANOVA).

Supplementary Figure 12.

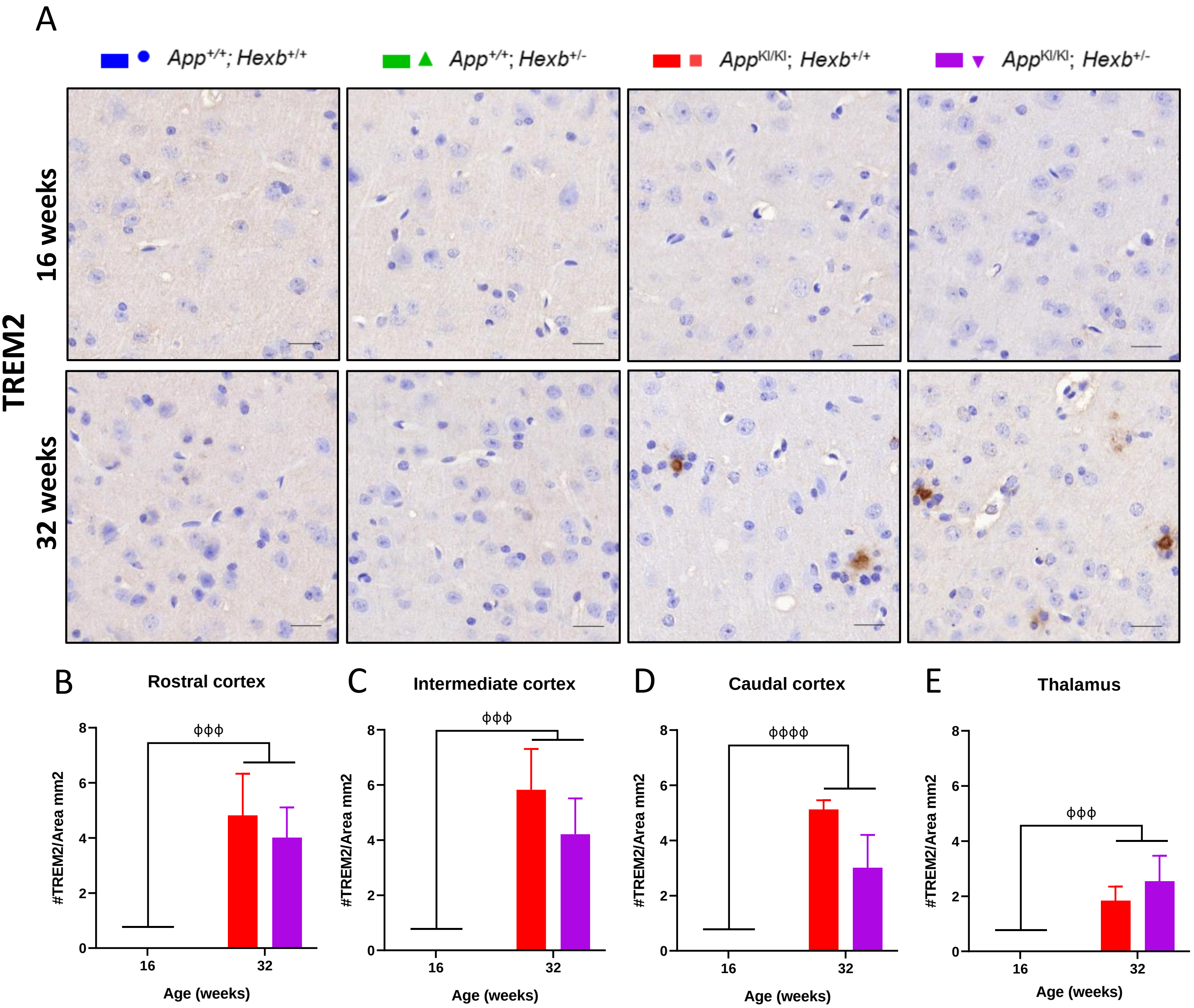

Supplementary Figure 12 Triggering receptor expressed on myeloid cells 2 (TREM2) staining. Staining for TREM2 is shown in rostral cortex (A). Scale bars = 20 µm. Quantification of the area of TREM2 staining in *App*$^{KI/KI}$; *Hexb*$^{+/+}$ and *App*$^{KI/KI}$; *Hexb*$^{+/-}$ mice at 16 and 32 weeks was performed in the rostral cortex (B), intermediate cortex (C), caudal cortex (D), and thalamus (E) n = 5 mice of mixed sex/group, error bars = ± SEM, ϕϕϕ $p<0.001$, ϕϕϕϕ $p<0.0001$ age effect (two-way ANOVA).

Supplementary Figure 13.

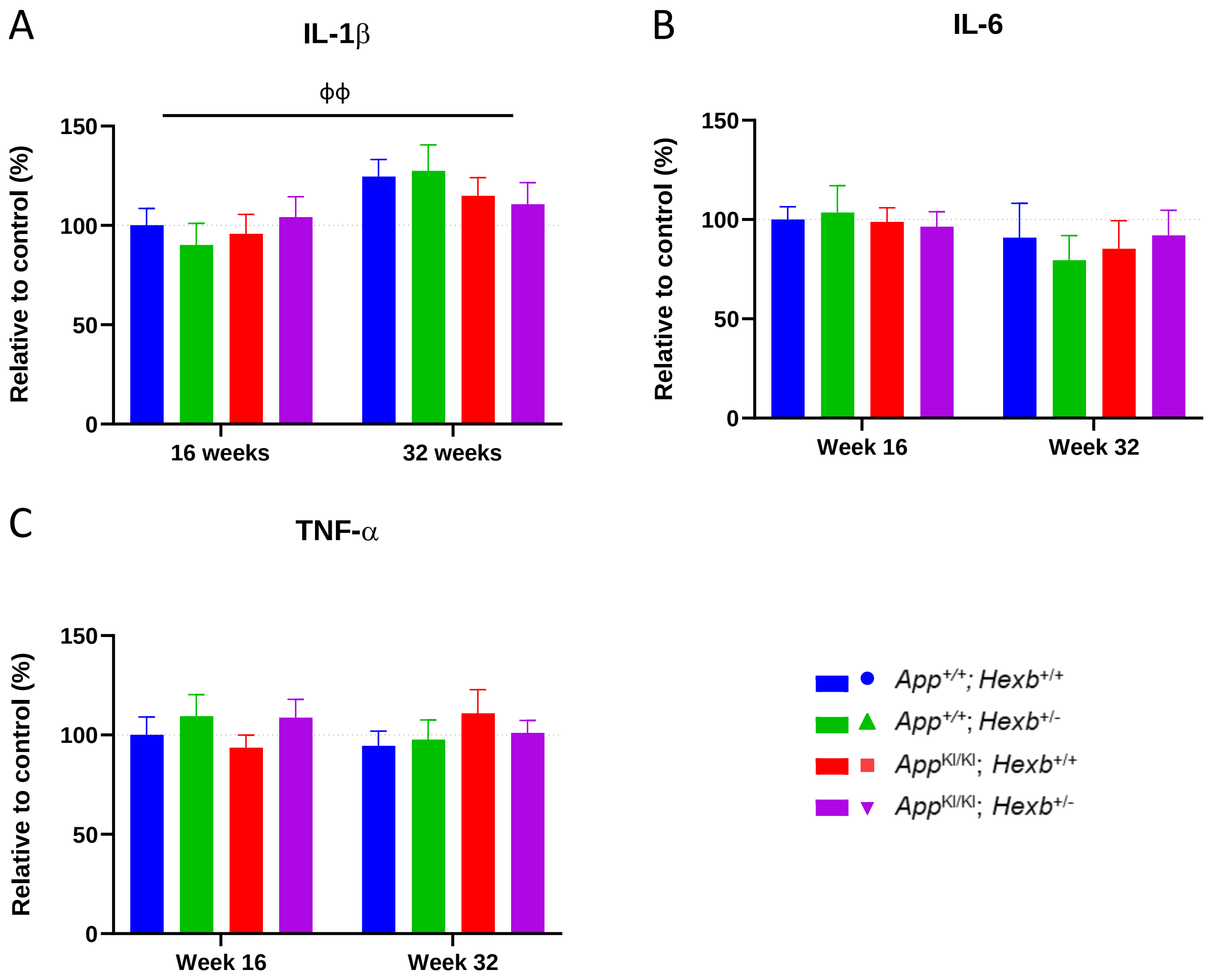

Supplementary Figure 13. Cytokine protein levels in mouse cortex. Interleukin-1β (IL-1β) (A), Interleukin-6 (IL-6) (B), and Tumor Necrosis Factor-α (TNF-α) (C) were measured with ELISA assays in cortical homogenates from *App*$^{+/+}$; *Hexb*$^{+/+}$, *App*$^{+/+}$; *Hexb*$^{+/-}$, *App*$^{KI/KI}$; *Hexb*$^{+/+}$ and *App*$^{KI/KI}$; *Hexb*$^{+/-}$ mice at 16 and 32 weeks. n=5-10 mice of mixed sex/group, results are mean ± SEM, ϕϕ p<0.01 age effect (2-step analysis: age, *App* genotype and *Hexb* genotype effects and age**App***Hexb* interaction analysed with a three-way ANOVA; then individual time points analysed with a two-way ANOVA).
