# Supplementary material for: Lysosomal gene Hexb displays haploinsufficiency in a knock-in mouse model of Alzheimer’s disease
Source: IBRO Neurosci Rep. 2022 Jan 20;12:131–41. doi: 10.1016/j.ibneur.2022.01.004 (PMC8819126; doi:10.1016/j.ibneur.2022.01.004)
Supplement: Supplementary file 3 — Supplementary material [file mmc3.pdf]

Analyte	Q1 Mass (Da)	Q3 Mass (Da)	Dwell (msec)	CE
GM3 (d18:1/18:0) [M-H]	1179.7	290.1	25	-66
D_3 GM3 (d18:1/18:0) [M-H] (internal standard)	1182.9	290.2	25	-66
GM2 (d18:1/18:0) [M-H]	1382.8	290.3	25	-80
D_3 GM2 (d18:1/18:0) [M-H] (internal standard)	1386.1	290.3	25	-80
GM1 (d18:1/18:0) [M-H]	1544.9	290.1	25	-93
D_3 GM1 (d18:1/18:0) [M-H] (internal standard)	1548.0	290.2	25	-93

Supplementary Table 1 Transition list for the analysis of gangliosides
